# nandb—number and brightness in R with a novel automatic detrending algorithm

**DOI:** 10.1093/bioinformatics/btx434

**Published:** 2017-07-11

**Authors:** Rory Nolan, Luis A J Alvarez, Jonathan Elegheert, Maro Iliopoulou, G Maria Jakobsdottir, Marina Rodriguez-Muñoz, A Radu Aricescu, Sergi Padilla-Parra

**Affiliations:** 1 Wellcome Trust Centre for Human Genetics, University of Oxford, Oxford, UK; 2 Department of Structural Biology, University of Oxford, Oxford, UK

## Abstract

**Summary:**

An R package for performing *number and brightness* image analysis, with the implementation of a novel automatic detrending algorithm.

**Availability and implementation:**

Available at https://github.com/rorynolan/nandb for all platforms.

**Contact:**

rnolan@well.ox.ac.uk or spadilla@well.ox.ac.uk

**Supplementary information:**

Supplementary data are available at *Bioinformatics* online.

## 1 Introduction

‘Number and brightness’ (N&B) (Digman *et al.*, 2008) is a fluorescence fluctuation spectroscopy technique to measure the average number and oligomeric state of *mobile* (freely diffusing) molecules in each pixel of an image. Define an *entity* as a set of molecules which are chemically bound (an entity can be a single molecule) and the *brightness* of an entity as the average number of photon detector counts it gives per unit time when in the illumination volume. Then, performing moment analysis of the pixel intensities over time, assuming molecular diffusion (i.e. assuming that the molecules are *mobile*), one finds that for a given pixel

(1) $$N\;=\frac{{\left\langle I\right\rangle }^{2}}{\sigma^{2}}=\frac{\epsilon n}{\epsilon +1}$$

(2) $$B=\frac{\sigma^{2}}{\left\langle I\;\right\rangle }=\epsilon +1$$

where I is the intensity (measured in counts) and thus ⟨I⟩ is the average intensity over time, $\sigma^{2}$ is the variance of that intensity over time, ϵ is the brightness of the average-sized entity (measured in counts per frame), B is the ‘apparent brightness’, n is the average number of entities and N is the ‘apparent number’. The brightness ϵ of an entity is directly proportional to its oligomeric state. This is because a dimer will have twice as much fluorescent protein as a monomer, therefore it will give twice as many detector counts per unit time (and so on). In this way, it is possible to directly relate changes in brightness to changes in oligomeric state.

Take care to note that Equations (1) and (2) hold for mobile entities only, that immobile entities have brightness B=1, and that regions in which there are mobile and immobile entities will have brightnesses B which are a weighted average (with unknown weights) of B=1 and B=1+ϵ. This is a limitation however in these regions this technique still functions to detect *changes* in oligomeric state (low → high or high → low).

Here we present an R (R Core Team, 2016) package for performing N&B analysis. The package implements novel features such as automatic detrending of image series (to correct for bleaching), creation of N&B videos (time-series of N&B images) and functions to perform N&B analyses in high-throughput (on many files in parallel).

## 2 Materials and methods

### 2.1 Regions of interest

**Fig. 1:**
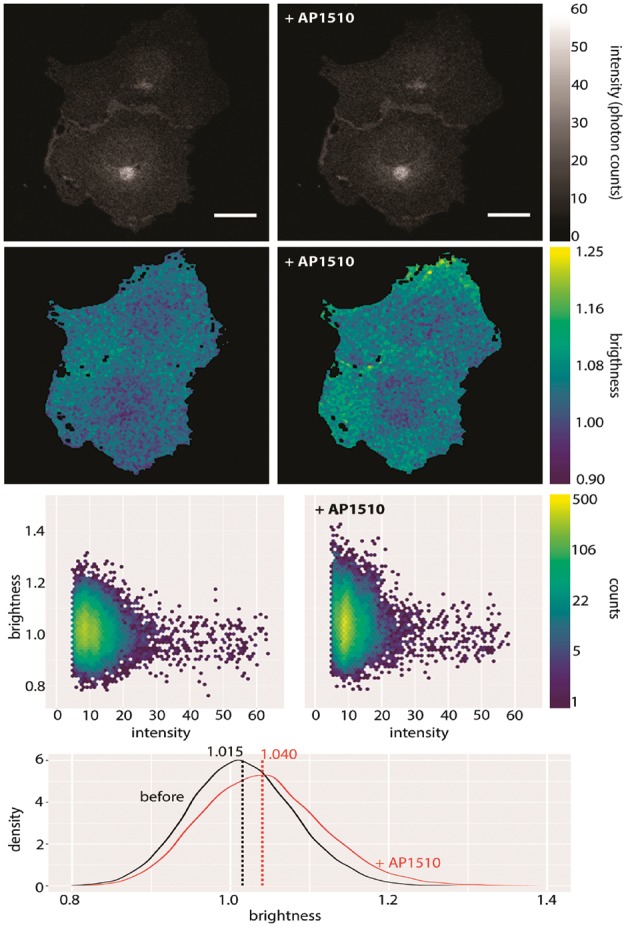
mClover-labelled myristoylated FKBP12 before and after application of 50nM AP1510. Shown here are intensity (first row), brightness (second row), a plot of intensity versus brightness (third row) and brightness histograms (fourth row). Notice how the change in brightness upon addition of the drug is seen most clearly by comparing the brightness histograms. The vertical lines in the histogram plot show the means of those histograms. Brightness here refers to B. Scale bar 20 µm

Prior to the performance of N&B calculations, the region(s) of interest of the image must be selected. For example, in Figure 1 , the region of interest is all the pixels where the cell is. It is important to exclude the *uninteresting* areas of the image (where the cell is *not*), as to include them would be to calculate N&B in regions where they cannot be interpreted in the same way. This would render incorrect calculations of quantities such as the mean brightness of the image, ruining the automatic detrending. One method of selecting regions of interest is to apply a threshold to exclude the low-intensity areas of the image. This package includes the option to use the *Auto Threshold* ImageJ plugin (Landini *et al.*, 2017) algorithms.

### 2.2 Detrending

Due to bleaching, the intensity of the image may decrease during the acquisition (as more frames are acquired). This artificially alters the variance of the intensities of each pixel and decreases their mean, spoiling the N&B calculations (Equations 1 and 2). Before doing these calculations, one may correct for this bleaching, i.e. one may *detrend* the image series. Digman *et al.* suggest the *exponential filtering* method of detrending (see Supplementary Material for more on exponential filtering). Whilst this is indeed a good detrending method, the question remains: how does one choose the exponential parameter τ for the filtering? Higher values of τ will filter out long-term trends whilst lower values will filter out short and long-term trends: as τ→ ∞, exponential filtering detrending does nothing at all, whereas as τ→ 0, it sets everything to the mean. With the ideal τ, the detrending will filter out trends that occur over the relatively long period of the acquisition (i.e. trends due to bleaching) but will not filter out the local (short-term) intensity fluctuations that are used in Equations (1) and (2). Previously, τ had to be chosen arbitrarily (e.g. Digman *et al.* use τ= 10). We have implemented a novel *automatic detrending* technique that is capable of choosing a suitable value of τ for each image. The idea is to re-simulate the image series as having come from immobile particles only, preserving the mean of each frame. Then, using our knowledge that immobile particles must give mean brightness B =1, we choose the τ for which an exponential filtering detrend with this parameter results in the simulated image series satisfying mean$\left(B\right)=1$ [see Supplementary Material].

Unsurprisingly, even the detrending routine with the correct τ does not succeed in perfectly restoring the image series to how it would be in the absence of bleaching. Through simulation and real data, we have found that even if detrending is to be employed, one should try to restrict bleaching in image series as much as possible (by reducing laser power) and ensure that bleaching removes <25% of overall intensity (see Supplementary Material). This reduction in laser power will reduce brightnesses, however the automatic detrending routine excels at dealing with low brightnesses (even as low as B=1.05 after detrending), so this is generally not a problem.

This package provides the convenience function CorrectForBleachingFolder(tau=“auto”) which can automatically detrend all images in a folder in parallel. This is particularly useful for those who wish to use the automatic detrending algorithm but wish to carry out the rest of their analysis in a software other than nandb.

### 2.3 Number and brightness

Having followed these necessary pre-processing steps, one is now ready to perform N&B computations. This consists of simply applying formulae 1 and 2, pixel by pixel, to an image series. This functionality is provided via the Number() and Brightness() functions respectively. It is important to note that, in the nandb package, ‘number’ and “brightness” refer to N and B respectively (and *not* to n and ϵ). Also provided are functions NumberTxtFolder() and BrightnessTxtFolder() for performing these analyses on all of the image series in a folder in parallel. Since calculated number (N) and brightness (B) are real numbers (and not necessarily integers), the N&B images cannot be saved as tiff files (tiffs store integers only). This package saves these as csv files instead; these can be opened directly in imaging applications such as ImageJ. Metadata such as the detrending parameter used are recorded in the output file names. For a comparison of this software with other suites of N&B software, see the Supplementary Material.

### 2.4 FKBP12: Example of dimerization

Myristoylated FKBP12 is known to dimerize upon addition of the drug AP1510 (Amara *et al.*, 1997). We tested this in 20 Cos7 cells with mClover-labelled FKBP12. We found a brightness increase in ϵ of ∼1.6-fold, indicating that dimerization had occurred, but that not all of the protein had dimerized (as would be indicated by a 2-fold increase). Figure 1 shows the results for an example pair of cells. See Supplementary Material for full results.

## 3 Conclusion

nandb is an R package for performing ‘N&B’ image analysis. It includes a novel technique for automatically detrending image series affected by bleaching.

## Funding

This work has been supported by the Wellcome Trust (105278 to R.N., 203141 to S.P.), a Marie-Curie postdoctoral fellowship (FP7-328531 to J.E.) and the Medical Research Council (L009609 to A.R.A.).

*Conflict of Interest*: none declared.

## Supplementary Material

Supplementary Data
