# Supplementary material for: nandb—number and brightness in R with a novel automatic detrending algorithm
Source: Bioinformatics. 2017 Jul 11;33(21):3508–10. doi: 10.1093/bioinformatics/btx434 (PMC5860167; doi:10.1093/bioinformatics/btx434)
Supplement: Supplementary Data [file btx434_supp.pdf]

Supplementary Material

Rory Nolan, Luis A.J. Alvarez, Jonathan Elegheert, Maro Iliopoulou,
G. Maria Jakobsdottir, Marina Rodriguez-Muñoz,
A. Radu Aricescu and Sergi Padilla-Parra.

Contents

1 Exponential Filtering Detrending 2
1.1 Exponential Smoothing/Filtering . . . 2
1.2 Exponential Filtering Detrending . . . 2
1.3 Edges . . . 3
1.3.1 Extending the Series . . . 3
1.3.2 Neighbour-Smoothing and Mean Adjustment . . . 4
1.3.3 Visualising the Edge Effect . . . 4

2 Adaptive Detrending 5
2.1 Simulating the Image Series . . . 5
2.1.1 The Image Simulation Algorithm . . . 6
2.2 Finding τ . . . 6
2.3 The Box-Car . . . 6
2.4 Other Methods . . . 7

3 Myr-mClover-FKBP12 Construct Design 7

4 Cell Culture and Transfection 7

5 Imaging 7

6 FKBP12 Brightness Statistics 8

7 Simulations 10

8 Visualising Bleaching Correction 13

9 A Comparison of Number and Brightness Softwares 13

A Appendix 16
A.1 FKBP Results . . . 16
A.1.1 FKBP Full Results . . . 16
A.1.2 FKBP Brightness Fold Changes . . . 19
A.2 Simulations . . . 20
A.2.1 Performing the Simulations in SimFCS . . . 20
A.2.2 Simulation Full Results . . . 21
A.2.3 Simulation Results Summary . . . 30
A.2.4 Oligomeric State Results Within the Same Bleaching Regime . . . 33
A.3 Reproducibility . . . 35
A.3.1 Reproducing the Main Figure in the Paper . . . 35
A.3.2 Reproducing the FKBP Data . . . 35
A.3.3 Reproducing the Simulated Data . . . 35
A.3.4 Reproducing the Figure for Visualising Exponential Smoothing Edge Correction (section 1.3.3) . . . 35
A.3.5 Reproducing the Data for Visualising Bleaching Correction (section 8) . . . 36

1 Exponential Filtering Detrending

Note. In what follows:

- We present a generalized (*bi*-directional in time) version of the exponential filter described in [1]. If one hears the term *exponential smoothing/filtering* outside of the context of this paper, the description detailed in [1] should be assumed, unless otherwise stated.
- We will be discussing *time series*. Keep in mind that for an image series where the images are of the same space taken one after the other, each pixel is its own time series whereby the value at time t is the intensity value for that pixel in the frame acquired at that time t.

1.1 Exponential Smoothing/Filtering

In exponential *smoothing*, the idea is to *smooth* a time series by replacing each observation by a weighted average of that observation and the ones close to it. In this way, long-term trends are preserved, but local variation is said to be *filtered* away. Hence, exponential smoothing and exponential filtering refer to the same process.

Suppose that the time series y consists of N observations $y = \{y_i\}_{i=1,\ldots,N}$ taken at times $\{t_i\}_{i=1,\ldots,N}$. Then for time $t \in \{t_i\}_{i=1,\ldots,N}$, the corresponding exponentially smoothed estimates $\tilde{y} = \{\tilde{y}_i\}_{i=1,\ldots,N}$ are defined by

$$\tilde{y}_i \equiv \frac{\sum_{j=1}^{N} e^{-|t_i - t_j|/\tau} y_j}{\sum_{j=1}^{N} e^{-|t_i - t_j|/\tau}} \tag{1}$$

Now, if we measure time in units of frames (and the frames are captured at regular intervals), then we have $t_i = i \; \forall i$ (the first frame is $t = 1$, the second frame is $t = 2$ and so on). This gives

$$e^{-|t_i - t_j|/\tau} = e^{-|i-j|/\tau} \tag{2}$$

and thus

$$\tilde{y}_i = \frac{\sum_{j=1}^{N} e^{-|i-j|/\tau} y_j}{\sum_{j=1}^{N} e^{-|i-j|/\tau}} \tag{3}$$

Defining weights $w_{ij}(\tau) \equiv e^{-|i-j|/\tau}$, we get

$$\tilde{y}_i(\tau) = \frac{\sum_{j=1}^{N} w_{ij}(\tau) y_j}{\sum_{j=1}^{N} w_{ij}(\tau)} \tag{4}$$

which emphasises the fact that these weights are functions of τ and hence so are the smoothed $\tilde{y}_i$.

Note. It is imperative that if you use these units (frames), that τ is then measured in these units too.

1.2 Exponential Filtering Detrending

The idea of smoothing is to remove (*smooth out*) noise from our observation. That is, we believe that each data point y_i contains some interesting part α_i plus some noise ϵ_i:

$$y_i = \alpha_i + \epsilon_i \tag{5}$$

We hope that $\tilde{y}_i$ is a good approximation for α_i:

$$\tilde{y}_i \approx \alpha_i \tag{6}$$

Conversely, if we are more interested in ϵ than α, we can still use our estimate $\tilde{y}_i \approx \alpha_i$ as follows:

$$\begin{aligned} y_i = \alpha_i + \epsilon_i \Longrightarrow y_i &\approx \tilde{y}_i + \epsilon_i && \text{(by } \tilde{y}_i \approx \alpha_i\text{)} \\ \Longrightarrow \epsilon_i &\approx y_i - \tilde{y}_i && (7) \end{aligned}$$

So now given our observation y_i and our smoothed $\tilde{y}_i$, we can approximate ϵ_i as

$$\hat{\epsilon}_i = y_i - \tilde{y}_i \approx \epsilon_i \tag{8}$$

So now we have approximations $\hat{\epsilon}_i$ of the noise terms ϵ_i, to do with as we please.

Suppose we believe that (ideally) our time series should not contain any trends, i.e. that α should remain constant—we should have $\alpha_i = \alpha_0 \; \forall i$ for some α_0—and that the time-series should be just fluctuations around this α_0 due to varying ϵ_i. Then, knowing the $\hat{\epsilon}_i$ from above, we can reconstruct the time series as we think it *should* be using

$$\hat{y}_i = \alpha_0 + \hat{\epsilon}_i \tag{9}$$

where $\hat{y}_i$ are the points of the reconstructed series. The question remains: how do we choose the value α_0? It makes sense that the time-series be thought of as fluctuating around its mean value. Hence, it makes sense to set α_0 to the mean of the observations y_i:

$$\alpha_0 = \bar{y} = \frac{1}{N}\sum_{i=1}^{N} y_i \tag{10}$$

The series $\hat{y}$ has no overall trend and is just fluctuations around α_0 due to $\hat{\epsilon}$, so we say $\hat{y}$ is the *detrended* version of y. This is *exponential filtering detrending*: the use of exponential filtering to detrend a time series.

Note. The values $\hat{\epsilon}_i$ depend on the values $\tilde{y}_i$ of the smoothed time series, therefore the values $\hat{y}_i$ of the detrended time-series depend on the values $\tilde{y}_i$ of the smoothed time series. In turn, the $\tilde{y}_i$ depend on the choice of the parameter τ. Hence, the values $\hat{y}_i$ of the detrended time-series depend on the choice of τ.

1.3 Edges

For time series with a trend, there is a problem at the edges since the calculation of the smoothed $\tilde{y}$ values here are done from points all on one side of the point of interest. For example, for the first point of the series, the smoothed value is calculated from points $1, 2, 3, \ldots$, but there are no points $0, -1, -2, \ldots$ on the other side. Hence, if there is an upward trend, the smoothed value for point 1 will be pulled up by points $2, 3, \ldots$, but not down by the preceding points because there are none. There's nothing special about the edges, they are just the points where we started and stopped recording, therefore this edge effect is unwanted. To remove this edge effect from the smoothing process, we extend the time series in both directions with a non-fitting approach, smooth the whole extended series and then remove the parts of the extended series which were added during the extension. In the smoothed extended series, the edge-effected parts are its edges (and not the edges of the original series), i.e. the edge-effected parts are in the extended sections. Therefore when the extended sections are removed, what is left is not edge-effected.

1.3.1 Extending the Series

The ith point y_i in an N-point time-series $y = \{y_i\}_{i=1,\ldots,N}$ can be *reflected* in the points on the left of it to become points $i - 2(i-1) = 2 - i$ (so point 2 reflects to point 0, point 3 reflects to point -1 and so on) and on the right of it to become points $i + 2(N - i) = 2N - i$ (so point $N-1$ reflects to point $N+1$, point $N-2$ reflects to point $N+2$ and so on) respectively.

To reflect point i to the left, take points $1, \ldots, i-1$ and calculate their median $m_{i_{\text{left}}}$. The mean distance of the points used to calculate this median from point i is

$$\text{mean}(i-1, i-2, \ldots, 2, 1) = 1 + \text{mean}(i-2, i-1, \ldots, 2, 1, 0) = 1 + \tfrac{1}{2}(i-2) = \tfrac{1}{2}i \qquad (11)$$

We want to reflect it to point $2-i$ which is a distance $i-(2-i) = 2(i-1)$. Since the *distance* of the point to reflect it in is $\frac{1}{2}i$, the value of the reflected point should be

$$y_{2-i} = y_i + \frac{2(i-1)}{\frac{1}{2}i}(m_{i_{\text{left}}} - y_i) = y_i + 4\left(\frac{i-1}{i}\right)(m_{i_{\text{left}}} - y_i) \qquad (12)$$

To reflect point i to the right, take points $i+1, i+2, \ldots, N$ and calculate their median $m_{i_{\text{right}}}$. The mean distance of the points used to calculate this median from point i is

$$\text{mean}(1, 2, \ldots, N-i) = 1 + \text{mean}(0, 1, \ldots, N-i-1) = 1 + \tfrac{1}{2}(N-i-1) \qquad (13)$$

We want to reflect it to point $2N-i$ which is a distance $(2N-i)-i = 2(N-i)$. Since the *distance* of the point to reflect it in is $1 + \frac{1}{2}(N-i-1)$, the value of the reflected point should be

$$y_{2N-i} = y_i + \frac{2(N-i)}{1 + \frac{1}{2}(N-i-1)}(m_{i_{\text{right}}} - y_i) \qquad (14)$$

1.3.2 Neighbour-Smoothing and Mean Adjustment

The *extended* part of the series tends to be more varied (more spontaneous peaks) than the original part, so it is neighbour-smoothed by taking each point as the average of itself and its two nearest neighbours. The edge points only have one nearest neighbour so they are neighbour-smoothed using the formula $\frac{1}{3}(2(\text{edge point}) + \text{nearest neighbour})$.

This *extended* series isn't guaranteed to have the same mean as the original, however it's important that it does to preserve the property of exponential smoothing that as $\tau \to \infty$, the smoothed series tends towards a series in which every point is equal to the mean of the original series. To do this, we adjust the points of the extended series to bring them closer to this desired mean such that the overall mean of the extended series is equal to that of the original series in such a way that points 0 and $N+1$ aren't moved at all, points -1 and $N+2$ are moved a distance d, points -2 and $N+3$ are moved a distance $2d$, points -3 and $N+4$ are moved a distance $3d$ and so on. This method of adjusting the extended parts of the series preserves continuity at the points where the original series meets the extended series.

1.3.3 Visualising the Edge Effect

Let's take a look at what happens when this edge correction is and is not applied. Figure S1 shows the exponentially smoothed version of the series $\{0, 1, 2, \ldots, 99, 100\}$ (which is a perfectly straight line) with and without edge correction. The smoothing parameter used is $\tau = 40$. We see that when the edge correction is not applied, values are pulled further towards the mean[1] (which is equal to 50 here) and the smoothed series takes on a sigmoidal shape. There is no good reason for a smoothing method to turn a straight line into a sigmoid[2], so the naive method is erroneous.[3]

[1]When edge correction is applied, values are still pulled towards the mean, as is normal when smoothing.

[2]We want the smoothing to make the series *smoother*, but not to change its nature (e.g. from linear to sigmoidal).

[3]In fact, even the corrected series is be slightly sigmoidal, but to a negligible degree.

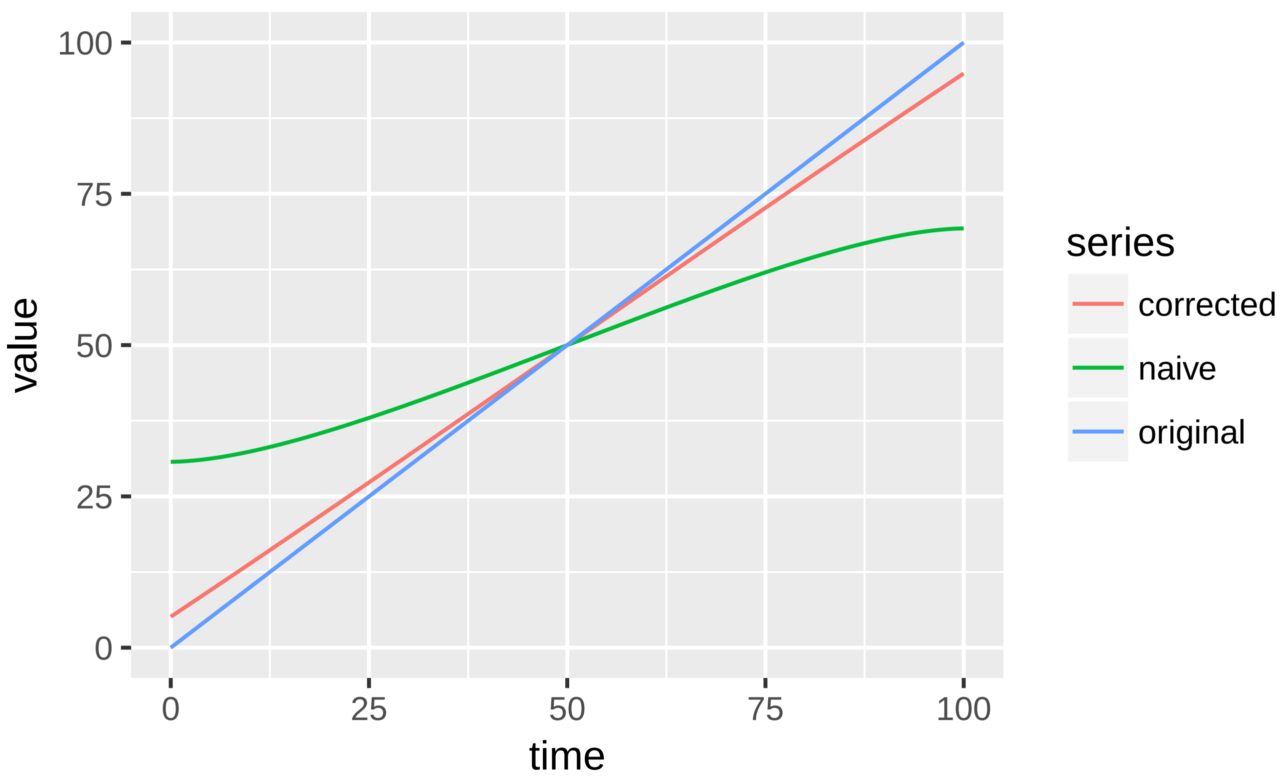

Figure S1: A plot of exponentially smoothed (with smoothing parameter $\tau = 40$) version of the series $\{0, 1, 2, \ldots, 99, 100\}$ with and without edge correction.

2 Adaptive Detrending

We would like to know how to choose a good value for τ. Once this choice is made, the detrending algorithm described above may proceed. Hence, if the choice of τ is automatic, then so is the whole detrend.

Given an image series of real experimental data, if we simulate an image series as having frames with the same means as the experimental frames, but as having come from immobile particles only, then this simulated series has the same amount of bleaching (as measured by the change in frame means) but we have the added information that—after correcting for bleaching—the brightnesses of the pixels of this simulated image series should have mean 1.[4] Hence, the choice of τ for the exponential filtering detrend for the simulated series should be the one that corrects it such that it has mean brightness 1.[5]

Since the simulated and real image series have the same amount of bleaching (as measured by the change in frame means), they warrant the same bleaching correction, i.e. the same choice of parameter τ. Thus, given that we know how to choose τ for the simulated series, we know how to choose it for the real data.

2.1 Simulating the Image Series

You wish to simulate n frames of p pixels each. What is p? Say the original frames have P pixels (e.g. $256 \times 256 = 65536$). You may specify a region of interest in this image, thereby you exclude

[4]We know that immobile particles should give mean brightness 1. This is crucial information that we get from replacing mobile particles with immobile ones; before doing this, there was a mix of mobile and immobile and all we knew was that the average brightness should be some value greater than or equal to 1.

[5]These simulations are random and hence each one will be different from the last. This introduces randomness into the solved-for value of τ, meaning that if we run the τ-finding routine twice, we could get slightly different values. This reflects a real uncertainty as to what the best choice of τ is.

some pixels and are left with p pixels where $p < P$ (the other pixels should be set to `NA`). If the whole image is of interest, then $p = P$.

The only criteria that we want our simulated image series to satisfy are:

1. The frames have the same number of pixels as the recorded frames (after selection of regions of interest).

2. They are simulated as having counts coming from immobile particles only.

3. The ith frame in the simulated series has the same mean intensity as the ith frame in the real data.

Here is how to satisfy those criteria:

1. If regions of interest are selected, pixels outside those regions should be set to `NA`. Then, to find the number of pixels p in a frame, just count the number of non-`NA` pixels in the frame after region of interest selection. If the whole original image is of interest, then it's just the number of pixels per frame in that image series.

2. Counts from stationary particles follow a Poisson distribution, so each pixel intensity value should be drawn from a Poisson distribution with some mean parameter λ.

3. Setting λ equal to the desired mean for the whole frame (so a different λ for each frame but the same λ for each pixel within a given frame) will ensure that the whole frame will have λ as its mean.[6]

2.1.1 The Image Simulation Algorithm

In brief, the steps to simulate an image are:

1. Count how many pixels p you want in each frame (it's the same for each frame).

2. Compute the means λ_i of each frame in the real data.

3. For each frame i, draw p times from a Poisson distribution with mean λ_i. These are your simulated pixel values for that frame.

The jth pixel in simulated frame i is the jth Poisson draw done with mean parameter λ_i. From here, you can carry on detrending and calculating brightness in the normal way.

2.2 Finding τ

Let's define a function f of τ:

$$f(\tau) = \text{mean brightness of simulated image series after detrending with parameter } \tau \qquad (15)$$

To find the best τ, we solve the equation $f(\tau) = 1$ for τ.

2.3 The Box-Car

Some prefer to use the *box-car* method (sometimes called the *running average* method) of detrending. We won't go into detail about this method, however it does require the user to specify the size of the box. The method used above to automatically decide upon the value of τ may be trivially adapted to select the size of the box. For a box of size l, replace the function f above with

$$f(l) = \text{mean brightness of simulated image series after detrending with box of size } l \qquad (16)$$

Then, to find the best l, choose the l for which $f(l)$ is closest to 1.

[6]In fact this mean may not be exactly λ, but the distance from λ will (by the central limit theorem) be negligible.

2.4 Other Methods

There are other methods (e.g. the polynomial fit detrend), but these all require the choice of some parameter γ (e.g. the degree of the polynomial). The method used above to automatically decide upon the value of the parameter τ may be trivially adapted to γ, whatever it may represent. For a detrending routine with parameter γ, use

$$f(\gamma) = \text{mean brightness of simulated image series after detrending with parameter } \gamma \tag{17}$$

Then, to find the best γ, choose the γ for which $f(\gamma)$ is closest to 1.

3 Myr-mClover-FKBP12 Construct Design

We constructed the fusion protein Myr-mClover-FKBP12 (figure S2 below), a membrane-associated version of the fluorescent protein mClover (Clover [2] monomerized by introducing the Ala to Lys mutation equivalent to Ala206Lys in GFP) that allows chemically induced dimerization (CID). To this end, we fused mClover (i) at its N-terminus to the myristoylation sequence of the proto-oncogene tyrosine-protein kinase Src from chicken (cSrc: Uniprot entry code P00523: Gly2-Leu18: GSSKSKPKDPSQRRRSL in single-letter amino acid code) and (ii) at its C-terminus to human peptidyl-prolyl cis-trans isomerase FKBP1A (also known as FKBP12 or FK506 binding protein: Uniprot entry code P62942: Gly2-Glu108). cSrc Gly2-Leu18 is predicted to form a potent myristoylation sequence, as judged by the MYR Predictor web application (`http://mendel.imp.ac.at/myristate/SUPLpredictor.htm` [3]). The sequence is myristoylated at Gly2, and myristoylation determines targeting to the plasma membrane. Additionally, the basic patch within Gly2-Leu18, rich in Lys and Arg residues, allows for favourable electrostatic interactions with the negatively charged phospholipid heads of the membrane. The Myr-mClover-FKBP12 construct was cloned into the pHLsec plasmid [4] and is expressed under control of a CAG promoter.

4 Cell Culture and Transfection

Cos-7 cells were grown using Dulbecco's Modified Eagle Medium (DMEM; Life Technologies) supplemented with 10% fetal bovine serum, 1% Penicillin-Streptomycin and 1% L-Glutamine (DMEMcomp). All cells were maintained in a 37°C incubator supplied with 5% CO_2. Cos-7 cells were seeded into μ-8 well slides (ibidi) at 60–70% confluence the day prior to transfection and incubated at 37°C. Plasmid pHLsec-Myr-mClover-FKBP12 was transfected using GeneJuice (Novagen) in accordance with the manufacturer's instructions. Phenol red DMEMcomp was removed from the cells 24 hours post transfection and replaced with DMEMcomp lacking phenol red for live cell imaging purposes.

5 Imaging

Cells transiently expressing myr-mClover-FKBP12 [4] were imaged before and after addition of AP1510 A/A Homodimerizer (Clontech Laboratories) at 37°C, in accordance with the manufacturers instructions. The imaging was performed on a Leica SP8 confocal microscope with a 63x/1.4 N.A. oil immersion objective and one HyD internal detector capable of single photon counting. The axial position of a specimen during acquisition was stabilized using the Adaptive Focus module. mClover was excited at 488nm and emitted light was collected at 500–550nm, using the white light laser at 10% power. For each cell, 50 frames were acquired at a rate of 3 frames per second with 2.43 μs pixel dwell time and pixel size 360 nm.

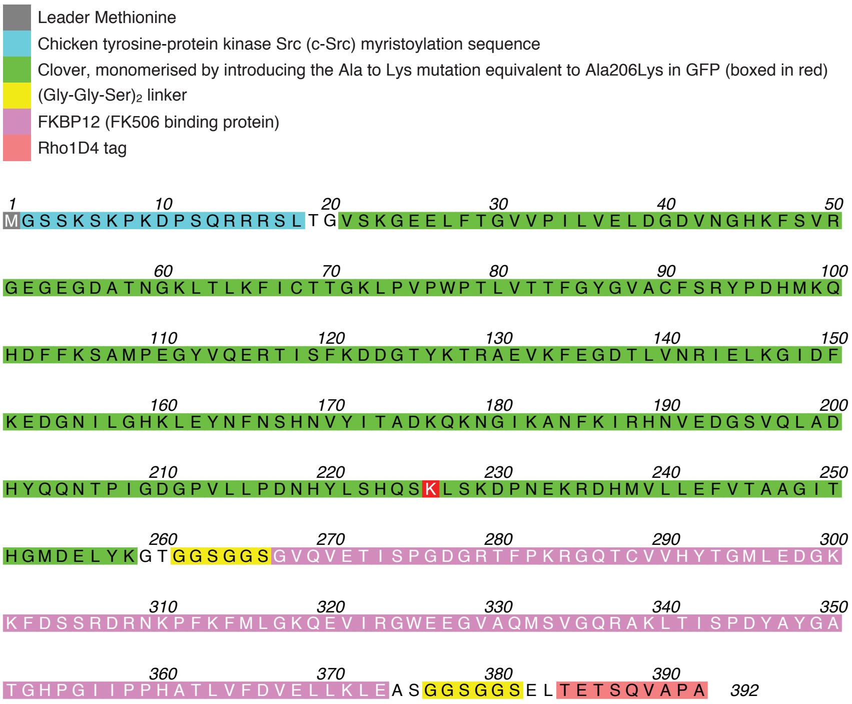

Figure S2: Sequence of the Myr-mClover-FKBP12 fusion protein. The chicken tyrosine-protein kinase Src (c-Src) myristoylation sequence is coloured in cyan, mClover is coloured in green, the two (Gly-Gly-Ser)2 linkers are coloured in yellow, FKBP12 is coloured in magenta and the Rho1D4 tag is coloured in salmon pink. The Clover monomerizing Ala to Lys mutation, equivalent to Ala206Lys in GFP, is boxed in red.

6 FKBP12 Brightness Statistics

Note. Henceforth, "FKBP" refers to FKBP12.

We imaged 20 cells before and after addition of AP1510 at concentrations of 0.5nM, 5nM, 12.5nM and 25nM (5 cells at each concentration), expecting to detect a dimerization of FKBP upon addition. We calculated brightness without detrend, with exponential filtering detrending with $\tau = 10$ and with the automatic detrending procedure described above. See the full results in appendix section A.1.1. We noted that at each concentration, the brightness was increased upon addition of the drug but that the different concentrations did not differ in their effects (no concentration produced significantly higher brightnesses than another). This can be seen in figure S3. Accordingly, we henceforth pool the cases of addition of these different concentrations of drug into one *after drug* category.

Figure S4 shows the fold change in brightness ϵ for each cell. This data is given in full in appendix section A.1.2. Due to the presence of outliers in this population of fold changes, the use of mean and standard deviation as summary statistics is inappropriate (e.g. for no detrend, most fold changes are in the region of 1.5, but some are at ≈ 7, so these would massively skew the mean). Instead we use the median as a robust measure of *typical* behaviour and the non-parametric bootstrapping technique (bias corrected, accelerated [5]) to measure uncertainty in the median. These statistics are given in table S1. We see that with $\tau = 10$, no increase in brightness ϵ is detected (indeed a decrease is detected). With no detrend, the median fold increase is 1.37, however no increase (fold increase of 1) is within the 95% confidence interval (0.97, 1.93). With automatic detrending, the median fold increase is 1.62, however a fold increase of 2 (indicating dimerization of all proteins, which is what one might expect) is within the 95% confidence interval (1.17, 2.65). A fold increase of 1.62 likely indicates that some but not all of the protein underwent dimerization. Additionally, labelled FKBP may interact with endogenous FKBPs, making dimers

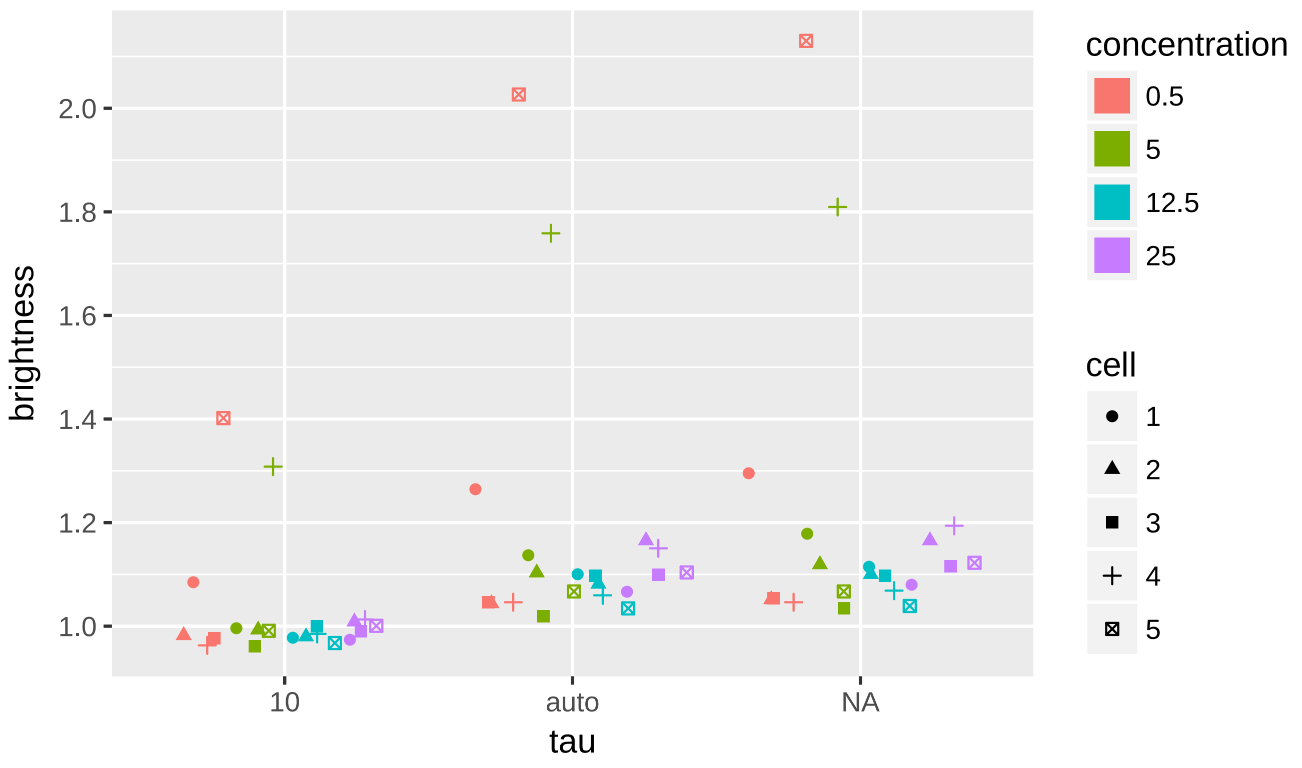

Figure S3: Distribution of brightnesses B for different detrending regimes and drug concentrations.

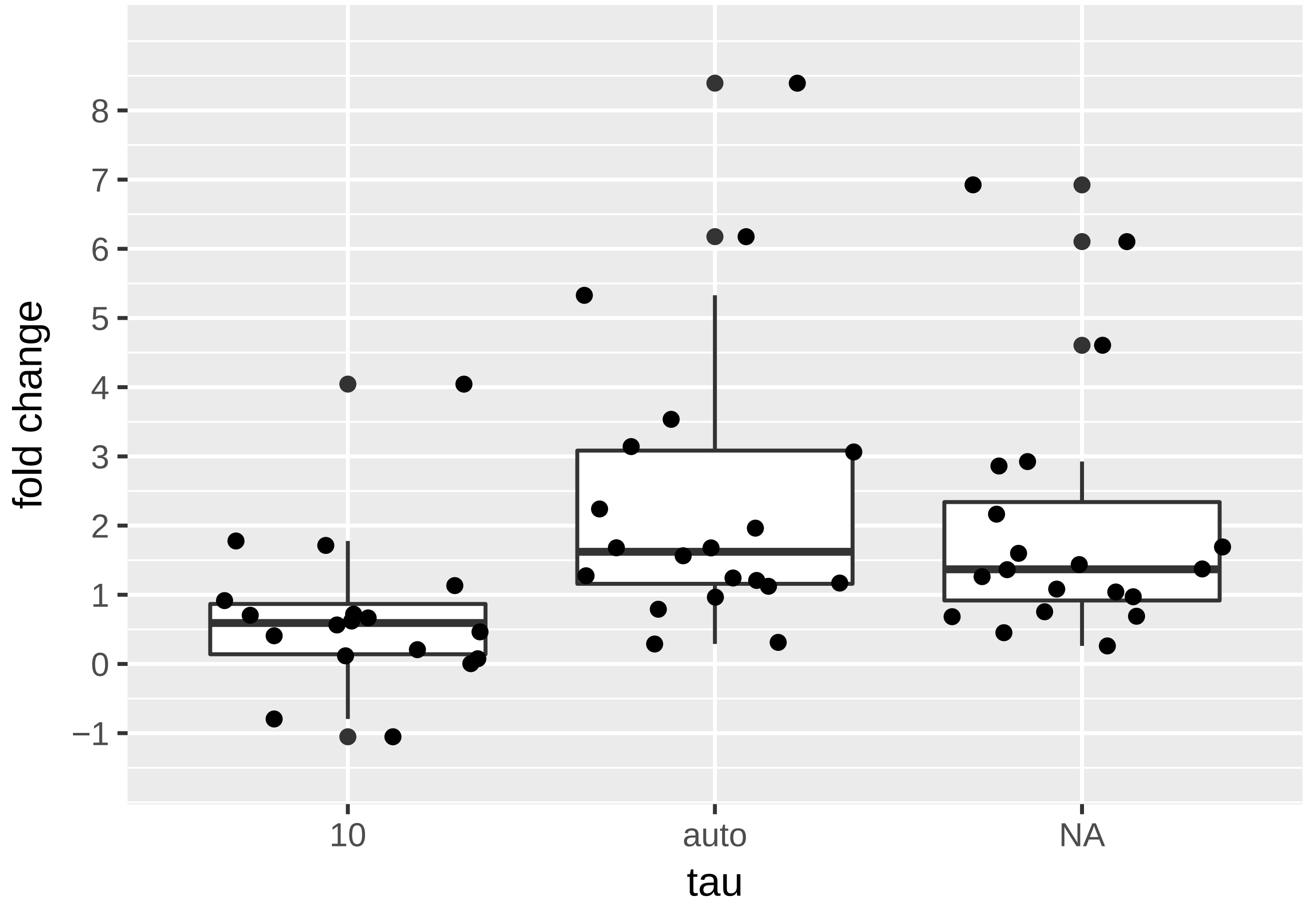

Figure S4: The fold changes in brightness ϵ upon addition of AP1510 drug shown for different detrending routines.

tau	Median fold change	lower	upper
10	0.59	0.14	0.81
auto	1.62	1.17	2.65
NA	1.37	0.97	1.93

Table S1: Median brightness ϵ fold changes (with upper and lower points of 95% confidence interval for median) in FKBP upon addition of AP1510 with different detrending routines.

appear as monomers [6]. Assuming that the drug functioned as expected, automatic detrending performed best here, as it was the technique that indicated most confidently that the drug did induce an increase in oligomeric state and it yielded a reasonable range of values for the scale of that increase. Notice that the 95% confidence intervals are quite wide, indicating that there is a lot of uncertainty as to what the actual *typical* fold change is. This uncertainty is real and comes from biological variability and an inherent uncertainty in the number and brightness technique. More replicates (more cells) would be required to decrease this uncertainty; one could also take more frames per cell (here we acquired 50 frames per cell). It is very important that these uncertainties be quoted when performing number and brightness studies (rather than simply concluding that the oligomeric state change indicated by the mean (or median) fold change is the one that happens every time). Robust statistics like those employed here are crucial to dealing properly with outliers, to which one is particularly susceptible when measuring fold changes.

7 Simulations

To assess the validity of the software, the efficacy of different detrending routines and the effect of different types of bleaching, we performed simulations of image series using the SimFCS [7] software (see appendix section A.2.1). We simulated a population with brightness $\epsilon = 0.95$ (i.e. $B = 1.95$) and then populations with brightnesses $\epsilon = 5 \times 0.95 = 4.75$ and $\epsilon = 10 \times 0.95 = 9.5$ to simulate monomers, 5mers and 10mers. We simulated three replicates of each condition with each image series having 500 frames. We then artificially bleached these with linear, exponential and power bleaching regimes with bleaching extents 0.5, 0.75, 0.9, 0.95 and 0.99. A bleaching extent of x means that the final frame has an average intensity of x times that of the first frame. A linear bleaching regime is such that the mean intensities of the frame decrease linearly. An exponential bleaching regime is such that the frame means decrease according to an exponential decay $y(t) = y_0 \exp(-\lambda t)$, so the decrease is more severe at the start than at the end. A power regime decay is a more extreme version of an exponential decay where $y(t) = y_0^{\lambda t}$. These decays are depicted in figure S5. The synthetic bleaching was performed by randomly removing counts from a frame until it had the desired mean intensity.

We then processed all of these images and assessed their brightnesses with no detrend, $\tau = 10$ and the automatic detrending routine described in section 2. Full results are given in appendix section A.2.2. First, we found that the software was finding the correct brightness values on the unbleached data. For the bleached data, we found that the original brightnesses could not be recovered by detrending.

Detrending reliably decreases the brightness (compared to the raw data), since it preserves the mean $\langle I \rangle$ but decreases variance σ^2. Exponential filtering detrending works by detecting trends in the data. For high values of τ, only long-term trends are removed whereas for small τ, short and long-term trends are removed. The hope when correcting for bleaching is that only the longer-term trends caused by bleaching are removed and that the random trends that appear due to fluorescence fluctuation will be short-term enough to not be removed, but in reality, some of those are removed too, leading to an over-reduction in brightness. This is a caveat of the technique.

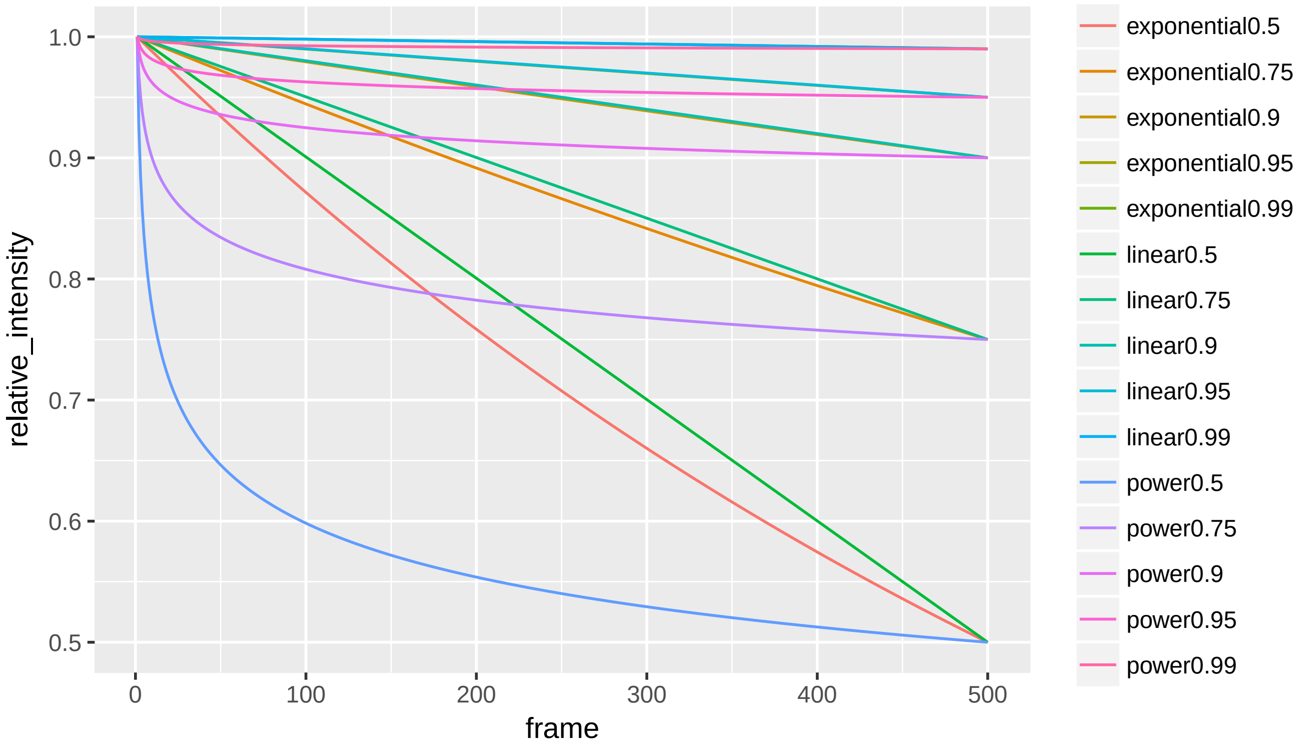

Figure S5: Decay regimes.

Figure S6 is a plot of the means with standard deviations[7] of the brightnesses of the replicates from each condition (every combination of simulated brightness, bleaching regime and detrending routine is its own *condition*). The data can be viewed in appendix section A.2.3. Importantly, even though the automatic detrending over-reduced the brightness, the change in oligomeric state could still be assessed with either no detrend at all or with automatic detrending if (i) the image series which were being compared bleached in the same manner[8] (see appendix section A.2.4) or (ii) the bleaching regime is gentle and the bleaching extent is not more extreme than 0.75 (a 25% decrease in intensity)[9].

To examine point (ii) further, we assess what happens with a set of acquisitions which is a mixture of the linear and exponential bleaching regimes with extents of 0.75 and 0.95, where we know that 5mers and 10mers should be detected (by 5-fold and 10-fold increases in brightness ϵ). Data is shown in table S2. Here $\tau = 10$ performs badly but even with no detrend and automatic detrending, we see that oligomeric states are under-estimated when there is a mix of bleaching regimes (5mers are mistaken for 4mers and 10mers are mistaken for 8 or 9mers). Indeed this problem is worse with automatic detrending than it is with no detrend at all.

So yes, on the simulated data, not detrending at all was a better strategy than using even the optimal exponential filtering detrend (the *automatic* detrend). The reason for this is as follows: Picture a 4mer with a bleaching extent of 0.75, this means that on average for each collection of 4 fluorescent proteins, one of them has bleached, making it look like a collection of 3 in the acquisition. This kind of error caused by bleaching is one which is not at all corrected by detrending routines. Let's call this a type A error. Detrending routines correct the series such that it has a stationary mean. Let's call the error caused by a non-stationary mean a type B error. For the case of no detrend, the non-stationary mean is not corrected for. Hence if we have bleaching, we have errors of type A and B. These actually compensate each other, since type A tends to decrease brightness and type B tends to increase it. Image series which are detrended do not suffer from

[7]Here, on simulated data, it is fine to use mean and standard deviation as summary statistics, since the simulated data is stable enough that there will be no massive outliers.

[8]This will never be the case in practice.

[9]This can be achieved in practice.

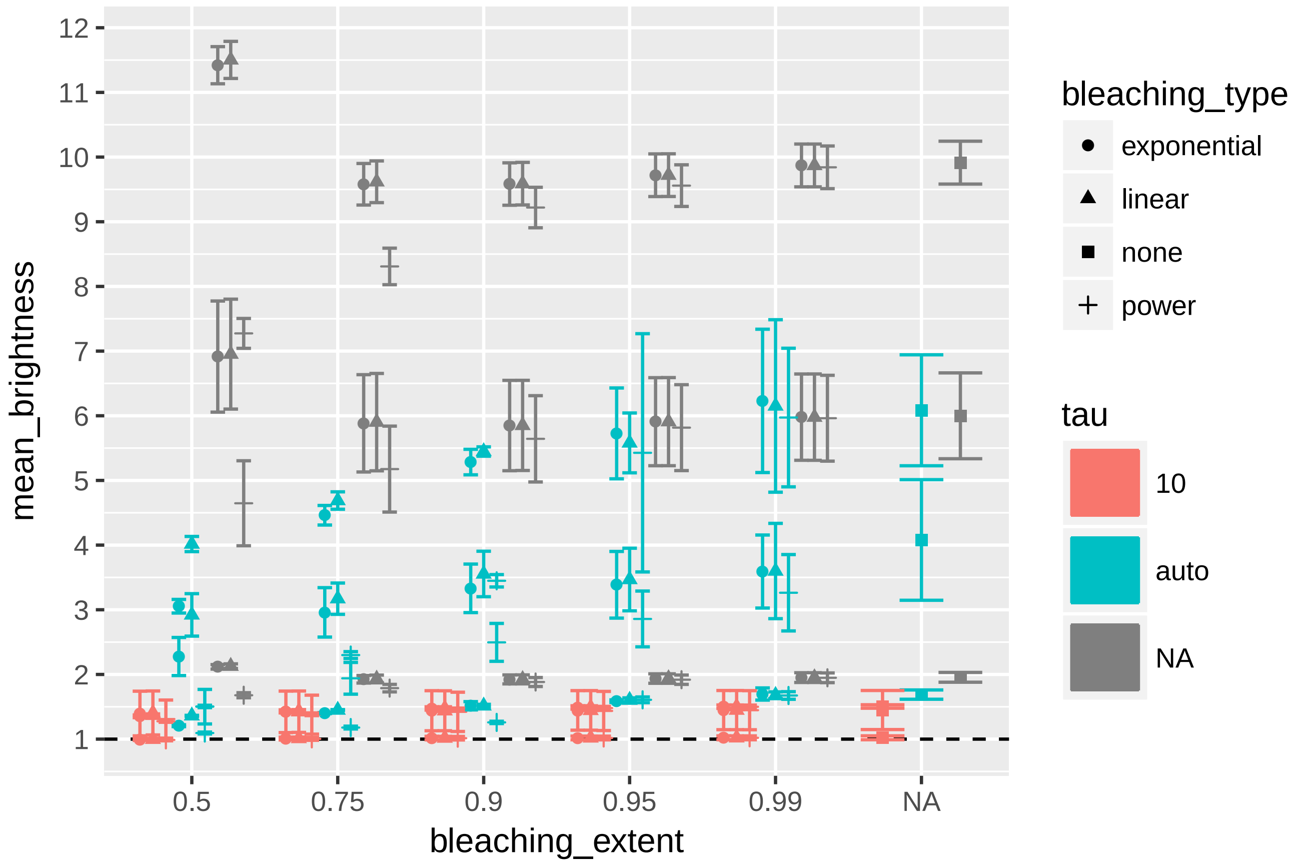

Figure S6: The brightnesses B of the simulated images with various detrending routines. Within a given τ (i.e. within a given colour), 3 distinct clusters of brightness, each with a •, ▲ and + indicates that three distinct oligomeric states have been found and that this identification was robust to changes in the detrending regime (linear, exponential or power). One can see here that we begin to lose this desired property for automatic detrending (blue) at 0.9 and that it is completely gone at 0.75.

oligomer	tau	Median	lower	upper
5mers	10	17.60	15.90	21.50
5mers	auto	4.41	4.08	4.72
5mers	NA	5.04	5.01	5.24
10mers	10	22.50	15.20	31.10
10mers	auto	8.19	7.78	8.49
10mers	NA	9.33	9.18	9.58

Table S2: Fold increases detected in brightness ϵ with a mixture of linear and exponential bleaching regimes with extents of 0.75 and 0.95. 95% confidence interval upper and lower limits shown. We know (since it is simulated data) that 5-fold and 10-fold increases should be detected.

type B errors (although there may be other errors introduced by the detrending), therefore the type A effect of underestimating brightness is not compensated for and detrended series can end up with conclusions further from the truth than series which have not been corrected for bleaching.

The simulated data teaches us that we should aim to restrict the bleaching and not bleach further than an end-point mean intensity of 75% of the starting mean intensity, and that we should also ensure that the bleaching is gradual (unlike the power bleaching regime). Also, having to detrend is fundamentally worse than not bleaching at all,[10] since bleaching leads us to underestimate changes in oligomeric state. This underestimation is not drastic and will be mostly negligible when trying to detect dimerization or even trimerization, but erroneous detection like that of 4mers instead of 5mers or 8mers instead of 10mers is a distinct possibility. Further improvements in detrending methods are required to reduce the possibilities of these errors.

From the discussion is section 6 above, it is evident that the automatic detrend algorithm was favourable for the real FKBP data, but here for the simulated data, detrending was not the best strategy. This is likely because the simulated data was bleached in a very even, random way, whereas for the FKBP acquisitions, some parts of the image bleached more than others. This will often be the case in practice, and when it is, the detrend-free approach does not work well.

To conclude, since the automatic detrending routine worked best for the real data and worked adequately (close to best) for the simulated data, it is the only sensible choice for real experiments. One should take care to bleach as little as possible, not bleach further than an end-point mean intensity of 75% of the starting mean intensity, and finally bear in mind that an under-estimation of oligomeric state change can be introduced by a variety of factors.

8 Visualising Bleaching Correction

Let us take a look at the image series of 500 frames[11] of a cell with the same FKBP system without drug. We examine the same image that appears in the main paper. We use a plot of frame mean intensities to visualise bleaching. Figure S7 shows the raw data (no detrend), $\tau = 10$ and automatic detrending ($\tau = 42$). Figure S8 compares $\tau = 10$ with the automatic detrend of $\tau = 42$. We see that both choices for τ succeed in making the mean intensity of the image series stationary (it starts and ends at ≈ 11, whereas before it declined from ≈ 12 to ≈ 10). In figure S8, we see that there is a lot more variance left in the $\tau = 42$ curve. From looking at both curves, there is no way to know which one is *better*. For this reason, visualisations such as this are not a good way to judge the merits of detrending algorithms, they are just a means to visualise their effects. A more nuanced argument like the one made with the simulations in section 7 is required to make such judgements. There it is shown how automatic detrending is superior to just picking a constant value of τ.

9 A Comparison of Number and Brightness Softwares

Table S3 gives a feature comparison of softwares available for Number and Brightness analysis. It includes the software presented in this paper (`nandb`, an R package), Imaging FCS [8], Leica's application note 40 [9], SimFCS [7] and Zeiss' ZEN Black [10]. We see that `nandb` is the only software to offer batch processing and automatic detrending. It is also the first text-based interface. This has the advantage of interoperability and mutability: the package is by default compatible with R's package ecosystem (facilitating interoperation with a plethora of other statistics and data processing tools) and the source code is easily altered. GUIs (graphical user interfaces) are easier

[10] It is unsurprising that having to detrend is worse than not bleaching at all. This tells us that even though correction for bleaching restores some useful properties of the image series, bleaching causes an irreversible loss of information.

[11] We took an acquisition of 500 frames since 50 is often too few to visually get a sense of bleaching.

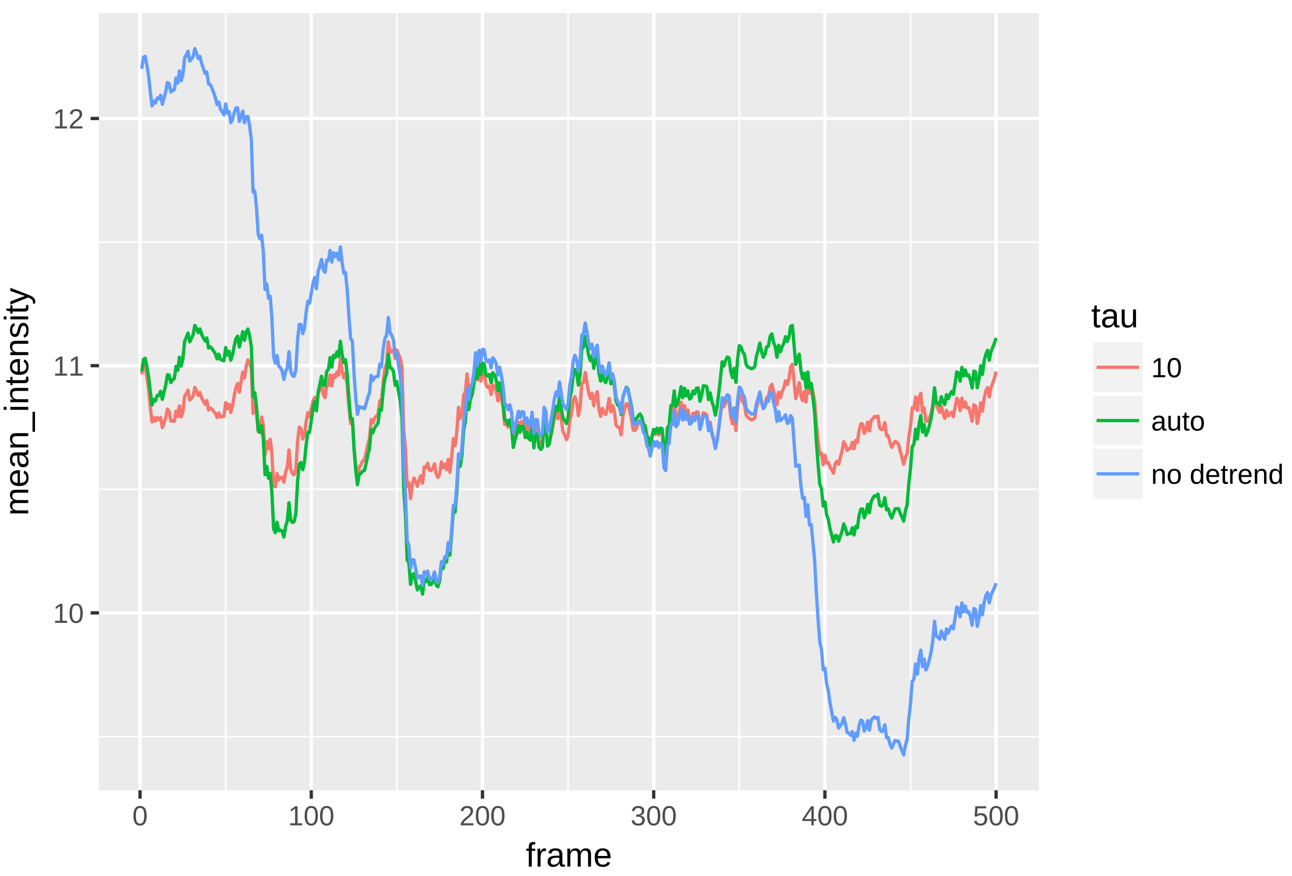

Figure S7: Visualising the detrending of the image series of cell 4.

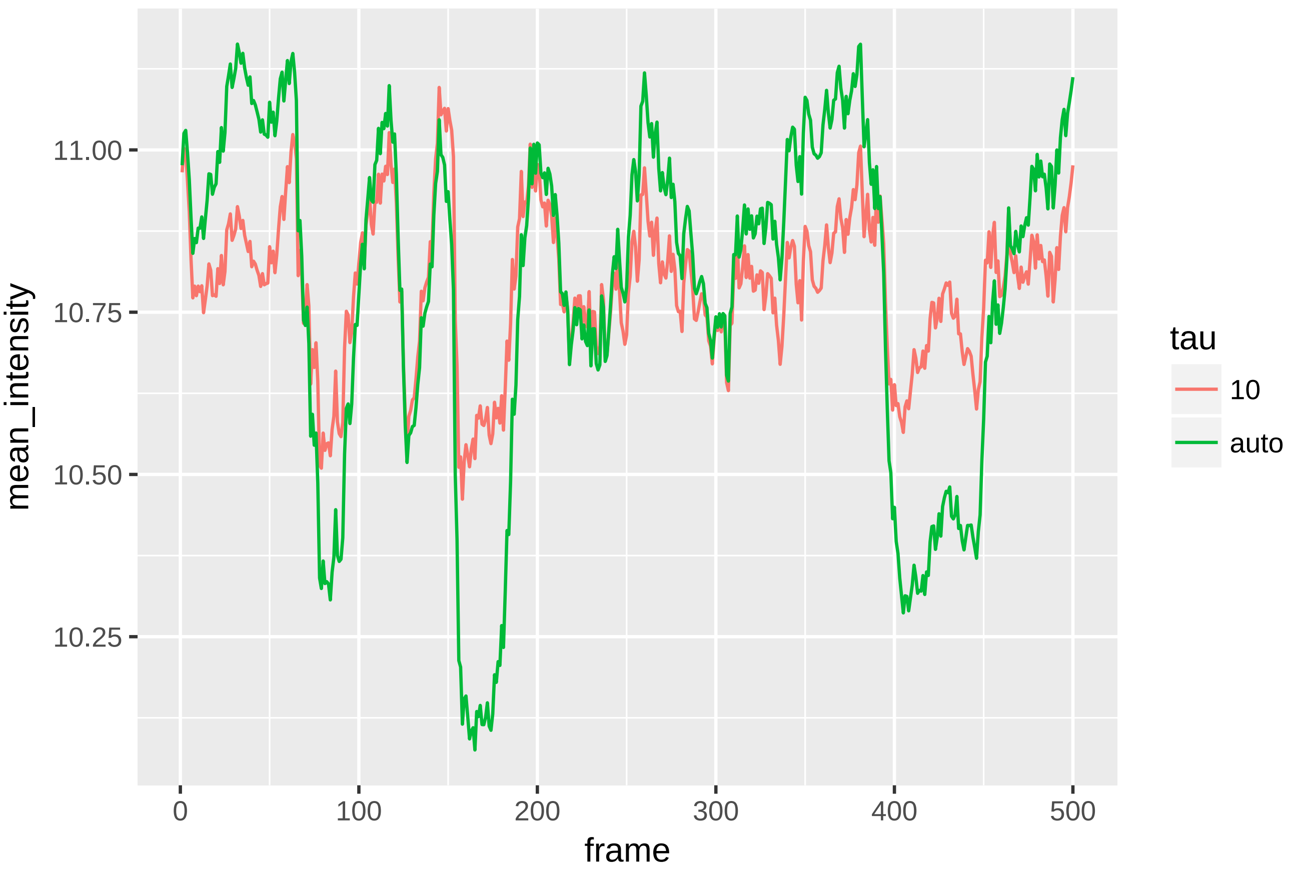

Figure S8: Comparing $\tau = 10$ and the automatically found $\tau = 42$ from figure S7.

	Imaging FCS	Leica	`nandb`	SimFCS	Zeiss
Automatic detrending	✗	✗	✓	✗	✗
Batch processing	✗	✗	✓	✗	✗
Detrending	✓	✗	✓	✓	✓
Free	✓	✓	✓	✗	✗
GUI	✓	✓	✗	✓	✓
Open source	✓	✓	✓	✗	✗
Read binary files	✓	✓	✓	✓	✗
Read TIFF files	✓	✓	✓	✓	✗
Save binary files	✓	✓	✓	✓	✓
Save text images	✓	✓	✓	✗	✓
Save TIFF files	✓	✓	✓	✗	✓
Textual	✗	✗	✓	✗	✗

Table S3: Features of Number and Brightness Softwares.

to navigate and provide excellent interactivity and visualisation tools which are not available in text-based softwares, however they are limited in the sense that the user cannot extend them to do anything beyond what was explicitly provided by their creator.

A Appendix

A.1 FKBP Results

A.1.1 FKBP Full Results

well	cell	drug	concentration	tau	auto_tau	brightness
1	1	before	0.00	10	FALSE	0.96
1	1	before	0.00	98	TRUE	1.02
1	1	before	0.00	NA	FALSE	1.04
1	1	after	25.00	10	FALSE	0.97
1	1	after	25.00	162	TRUE	1.07
1	1	after	25.00	NA	FALSE	1.08
1	2	before	0.00	10	FALSE	0.99
1	2	before	0.00	160	TRUE	1.10
1	2	before	0.00	NA	FALSE	1.12
1	2	after	25.00	10	FALSE	1.01
1	2	after	25.00	NA	TRUE	1.17
1	2	after	25.00	NA	FALSE	1.17
1	3	before	0.00	10	FALSE	0.98
1	3	before	0.00	269	TRUE	1.08
1	3	before	0.00	NA	FALSE	1.09
1	3	after	25.00	10	FALSE	0.99
1	3	after	25.00	189	TRUE	1.10
1	3	after	25.00	NA	FALSE	1.12
1	4	before	0.00	10	FALSE	1.00
1	4	before	0.00	142	TRUE	1.12
1	4	before	0.00	NA	FALSE	1.14
1	4	after	25.00	10	FALSE	1.01
1	4	after	25.00	126	TRUE	1.15
1	4	after	25.00	NA	FALSE	1.19
1	5	before	0.00	10	FALSE	1.01
1	5	before	0.00	102	TRUE	1.13
1	5	before	0.00	NA	FALSE	1.18
1	5	after	25.00	10	FALSE	1.00
1	5	after	25.00	150	TRUE	1.10
1	5	after	25.00	NA	FALSE	1.12
2	1	before	0.00	10	FALSE	0.97
2	1	before	0.00	NA	TRUE	1.02
2	1	before	0.00	NA	FALSE	1.02
2	1	after	12.50	10	FALSE	0.98
2	1	after	12.50	188	TRUE	1.10
2	1	after	12.50	NA	FALSE	1.11
2	2	before	0.00	10	FALSE	0.97
2	2	before	0.00	78	TRUE	1.05
2	2	before	0.00	NA	FALSE	1.10
2	2	after	12.50	10	FALSE	0.98
2	2	after	12.50	140	TRUE	1.08
2	2	after	12.50	NA	FALSE	1.10
2	3	before	0.00	10	FALSE	0.98
2	3	before	0.00	123	TRUE	1.05
2	3	before	0.00	NA	FALSE	1.06

2	3	after	12.50	10	FALSE	1.00
2	3	after	12.50	NA	TRUE	1.10
2	3	after	12.50	NA	FALSE	1.10
2	4	before	0.00	10	FALSE	0.96
2	4	before	0.00	105	TRUE	1.03
2	4	before	0.00	NA	FALSE	1.04
2	4	after	12.50	10	FALSE	0.99
2	4	after	12.50	138	TRUE	1.06
2	4	after	12.50	NA	FALSE	1.07
2	5	before	0.00	10	FALSE	0.96
2	5	before	0.00	128	TRUE	1.03
2	5	before	0.00	NA	FALSE	1.04
2	5	after	12.50	10	FALSE	0.97
2	5	after	12.50	136	TRUE	1.03
2	5	after	12.50	NA	FALSE	1.04
3	1	before	0.00	10	FALSE	0.97
3	1	before	0.00	93	TRUE	1.04
3	1	before	0.00	NA	FALSE	1.06
3	1	after	5.00	10	FALSE	1.00
3	1	after	5.00	143	TRUE	1.14
3	1	after	5.00	NA	FALSE	1.18
3	2	before	0.00	10	FALSE	0.97
3	2	before	0.00	129	TRUE	1.03
3	2	before	0.00	NA	FALSE	1.04
3	2	after	5.00	10	FALSE	0.99
3	2	after	5.00	170	TRUE	1.10
3	2	after	5.00	NA	FALSE	1.12
3	3	before	0.00	10	FALSE	0.98
3	3	before	0.00	133	TRUE	1.06
3	3	before	0.00	NA	FALSE	1.08
3	3	after	5.00	10	FALSE	0.96
3	3	after	5.00	88	TRUE	1.02
3	3	after	5.00	NA	FALSE	1.03
3	4	before	0.00	10	FALSE	1.01
3	4	before	0.00	91	TRUE	1.12
3	4	before	0.00	NA	FALSE	1.18
3	4	after	5.00	10	FALSE	1.31
3	4	after	5.00	186	TRUE	1.76
3	4	after	5.00	NA	FALSE	1.81
3	5	before	0.00	10	FALSE	0.99
3	5	before	0.00	111	TRUE	1.07
3	5	before	0.00	NA	FALSE	1.09
3	5	after	5.00	10	FALSE	0.99
3	5	after	5.00	NA	TRUE	1.07
3	5	after	5.00	NA	FALSE	1.07
4	1	before	0.00	10	FALSE	1.05
4	1	before	0.00	121	TRUE	1.22
4	1	before	0.00	NA	FALSE	1.27
4	1	after	0.50	10	FALSE	1.08
4	1	after	0.50	122	TRUE	1.26
4	1	after	0.50	NA	FALSE	1.30

4	2	before	0.00	10	FALSE	0.97
4	2	before	0.00	72	TRUE	1.04
4	2	before	0.00	NA	FALSE	1.07
4	2	after	0.50	10	FALSE	0.98
4	2	after	0.50	124	TRUE	1.04
4	2	after	0.50	NA	FALSE	1.05
4	3	before	0.00	10	FALSE	0.97
4	3	before	0.00	130	TRUE	1.03
4	3	before	0.00	NA	FALSE	1.04
4	3	after	0.50	10	FALSE	0.98
4	3	after	0.50	144	TRUE	1.05
4	3	after	0.50	NA	FALSE	1.05
4	4	before	0.00	10	FALSE	1.05
4	4	before	0.00	176	TRUE	1.16
4	4	before	0.00	NA	FALSE	1.18
4	4	after	0.50	10	FALSE	0.96
4	4	after	0.50	NA	TRUE	1.05
4	4	after	0.50	NA	FALSE	1.05
4	5	before	0.00	10	FALSE	0.99
4	5	before	0.00	142	TRUE	1.12
4	5	before	0.00	NA	FALSE	1.16
4	5	after	0.50	10	FALSE	1.40
4	5	after	0.50	221	TRUE	2.03
4	5	after	0.50	NA	FALSE	2.13

A.1.2 FKBP Brightness Fold Changes

well	cell	$\tau = 10$	$\tau =$ auto	no detrend
1	1	0.67	3.06	2.17
1	2	-1.05	1.68	1.44
1	3	0.46	1.17	1.26
1	4	4.05	1.27	1.36
1	5	0.08	0.79	0.68
2	1	0.72	5.33	6.10
2	2	0.62	1.68	1.04
2	3	0.01	1.96	1.60
2	4	0.41	2.24	1.69
2	5	0.92	1.12	0.97
3	1	0.12	3.54	2.86
3	2	0.21	3.14	2.93
3	3	1.78	0.31	0.45
3	4	31.80	6.18	4.61
3	5	1.13	0.96	0.75
4	1	1.71	1.21	1.08
4	2	0.56	1.24	0.69
4	3	0.70	1.56	1.37
4	4	-0.79	0.29	0.26
4	5	-44.48	8.39	6.92

Table A5: Fold changes in brightness ϵ for all FKBP cells upon addition of AP1510 with different detrending routines.

A.2 Simulations

A.2.1 Performing the Simulations in SimFCS

Simulations were performed using SimFCS software [7]. The parameters selected to recover three independent repeats per condition where: 400 molecules freely diffusing at $25\mu m^2/s$ for 1000 cycles. We picked different known brightnesses for monomers, 5mers and 10mers as specified in section 7.

A.2.2 Simulation Full Results

kmer	replicate	bleaching	tau	auto_tau	brightness
01	1	none	10	FALSE	1.02
01	1	none	731	TRUE	1.68
01	1	none	NA	FALSE	1.94
01	1	exponential0.5	10	FALSE	1.00
01	1	exponential0.5	63	TRUE	1.21
01	1	exponential0.5	NA	FALSE	2.11
01	1	exponential0.75	10	FALSE	1.01
01	1	exponential0.75	167	TRUE	1.40
01	1	exponential0.75	NA	FALSE	1.91
01	1	exponential0.9	10	FALSE	1.01
01	1	exponential0.9	302	TRUE	1.52
01	1	exponential0.9	NA	FALSE	1.91
01	1	exponential0.95	10	FALSE	1.01
01	1	exponential0.95	380	TRUE	1.57
01	1	exponential0.95	NA	FALSE	1.92
01	1	exponential0.99	10	FALSE	1.02
01	1	exponential0.99	696	TRUE	1.67
01	1	exponential0.99	NA	FALSE	1.93
01	1	linear0.5	10	FALSE	1.00
01	1	linear0.5	174	TRUE	1.36
01	1	linear0.5	NA	FALSE	2.12
01	1	linear0.75	10	FALSE	1.01
01	1	linear0.75	233	TRUE	1.45
01	1	linear0.75	NA	FALSE	1.92
01	1	linear0.9	10	FALSE	1.01
01	1	linear0.9	331	TRUE	1.53
01	1	linear0.9	NA	FALSE	1.91
01	1	linear0.95	10	FALSE	1.01
01	1	linear0.95	396	TRUE	1.57
01	1	linear0.95	NA	FALSE	1.92
01	1	linear0.99	10	FALSE	1.02
01	1	linear0.99	785	TRUE	1.69
01	1	linear0.99	NA	FALSE	1.93
01	1	power0.5	10	FALSE	0.99
01	1	power0.5	27	TRUE	1.09
01	1	power0.5	NA	FALSE	1.67
01	1	power0.75	10	FALSE	1.00
01	1	power0.75	41	TRUE	1.16
01	1	power0.75	NA	FALSE	1.78
01	1	power0.9	10	FALSE	1.01
01	1	power0.9	58	TRUE	1.24
01	1	power0.9	NA	FALSE	1.87
01	1	power0.95	10	FALSE	1.01
01	1	power0.95	447	TRUE	1.58
01	1	power0.95	NA	FALSE	1.90
01	1	power0.99	10	FALSE	1.02
01	1	power0.99	652	TRUE	1.66
01	1	power0.99	NA	FALSE	1.93

01	2	none	10	FALSE	0.99
01	2	none	467	TRUE	1.62
01	2	none	NA	FALSE	1.89
01	2	exponential0.5	10	FALSE	0.98
01	2	exponential0.5	59	TRUE	1.19
01	2	exponential0.5	NA	FALSE	2.08
01	2	exponential0.75	10	FALSE	0.99
01	2	exponential0.75	164	TRUE	1.41
01	2	exponential0.75	NA	FALSE	1.88
01	2	exponential0.9	10	FALSE	0.99
01	2	exponential0.9	173	TRUE	1.45
01	2	exponential0.9	NA	FALSE	1.86
01	2	exponential0.95	10	FALSE	0.99
01	2	exponential0.95	424	TRUE	1.59
01	2	exponential0.95	NA	FALSE	1.87
01	2	exponential0.99	10	FALSE	0.99
01	2	exponential0.99	473	TRUE	1.62
01	2	exponential0.99	NA	FALSE	1.89
01	2	linear0.5	10	FALSE	0.98
01	2	linear0.5	169	TRUE	1.37
01	2	linear0.5	NA	FALSE	2.09
01	2	linear0.75	10	FALSE	0.99
01	2	linear0.75	220	TRUE	1.46
01	2	linear0.75	NA	FALSE	1.88
01	2	linear0.9	10	FALSE	0.99
01	2	linear0.9	217	TRUE	1.49
01	2	linear0.9	NA	FALSE	1.86
01	2	linear0.95	10	FALSE	0.99
01	2	linear0.95	439	TRUE	1.60
01	2	linear0.95	NA	FALSE	1.87
01	2	linear0.99	10	FALSE	0.99
01	2	linear0.99	483	TRUE	1.62
01	2	linear0.99	NA	FALSE	1.89
01	2	power0.5	10	FALSE	0.97
01	2	power0.5	28	TRUE	1.08
01	2	power0.5	NA	FALSE	1.64
01	2	power0.75	10	FALSE	0.98
01	2	power0.75	45	TRUE	1.17
01	2	power0.75	NA	FALSE	1.74
01	2	power0.9	10	FALSE	0.99
01	2	power0.9	66	TRUE	1.26
01	2	power0.9	NA	FALSE	1.82
01	2	power0.95	10	FALSE	0.99
01	2	power0.95	414	TRUE	1.58
01	2	power0.95	NA	FALSE	1.86
01	2	power0.99	10	FALSE	0.99
01	2	power0.99	499	TRUE	1.62
01	2	power0.99	NA	FALSE	1.88
01	3	none	10	FALSE	1.05
01	3	none	696	TRUE	1.76
01	3	none	NA	FALSE	2.04

01	3	exponential0.5	10	FALSE	1.01
01	3	exponential0.5	56	TRUE	1.22
01	3	exponential0.5	NA	FALSE	2.15
01	3	exponential0.75	10	FALSE	1.03
01	3	exponential0.75	111	TRUE	1.39
01	3	exponential0.75	NA	FALSE	1.99
01	3	exponential0.9	10	FALSE	1.04
01	3	exponential0.9	274	TRUE	1.58
01	3	exponential0.9	NA	FALSE	2.00
01	3	exponential0.95	10	FALSE	1.05
01	3	exponential0.95	292	TRUE	1.60
01	3	exponential0.95	NA	FALSE	2.02
01	3	exponential0.99	10	FALSE	1.05
01	3	exponential0.99	901	TRUE	1.80
01	3	exponential0.99	NA	FALSE	2.03
01	3	linear0.5	10	FALSE	1.01
01	3	linear0.5	122	TRUE	1.35
01	3	linear0.5	NA	FALSE	2.17
01	3	linear0.75	10	FALSE	1.03
01	3	linear0.75	145	TRUE	1.44
01	3	linear0.75	NA	FALSE	2.00
01	3	linear0.9	10	FALSE	1.04
01	3	linear0.9	190	TRUE	1.52
01	3	linear0.9	NA	FALSE	2.00
01	3	linear0.95	10	FALSE	1.05
01	3	linear0.95	371	TRUE	1.64
01	3	linear0.95	NA	FALSE	2.02
01	3	linear0.99	10	FALSE	1.05
01	3	linear0.99	562	TRUE	1.72
01	3	linear0.99	NA	FALSE	2.03
01	3	power0.5	10	FALSE	1.00
01	3	power0.5	27	TRUE	1.11
01	3	power0.5	NA	FALSE	1.72
01	3	power0.75	10	FALSE	1.03
01	3	power0.75	40	TRUE	1.20
01	3	power0.75	NA	FALSE	1.85
01	3	power0.9	10	FALSE	1.04
01	3	power0.9	51	TRUE	1.28
01	3	power0.9	NA	FALSE	1.96
01	3	power0.95	10	FALSE	1.05
01	3	power0.95	438	TRUE	1.66
01	3	power0.95	NA	FALSE	2.00
01	3	power0.99	10	FALSE	1.05
01	3	power0.99	628	TRUE	1.74
01	3	power0.99	NA	FALSE	2.03
05	1	none	10	FALSE	1.80
05	1	none	321	TRUE	4.56
05	1	none	NA	FALSE	6.75
05	1	exponential0.5	10	FALSE	1.79
05	1	exponential0.5	67	TRUE	2.62
05	1	exponential0.5	NA	FALSE	7.89

05	1	exponential0.75	10	FALSE	1.79
05	1	exponential0.75	127	TRUE	3.40
05	1	exponential0.75	NA	FALSE	6.74
05	1	exponential0.9	10	FALSE	1.79
05	1	exponential0.9	157	TRUE	3.76
05	1	exponential0.9	NA	FALSE	6.64
05	1	exponential0.95	10	FALSE	1.79
05	1	exponential0.95	183	TRUE	3.97
05	1	exponential0.95	NA	FALSE	6.68
05	1	exponential0.99	10	FALSE	1.80
05	1	exponential0.99	232	TRUE	4.24
05	1	exponential0.99	NA	FALSE	6.74
05	1	linear0.5	10	FALSE	1.79
05	1	linear0.5	135	TRUE	3.29
05	1	linear0.5	NA	FALSE	7.92
05	1	linear0.75	10	FALSE	1.79
05	1	linear0.75	133	TRUE	3.45
05	1	linear0.75	NA	FALSE	6.76
05	1	linear0.9	10	FALSE	1.79
05	1	linear0.9	181	TRUE	3.90
05	1	linear0.9	NA	FALSE	6.64
05	1	linear0.95	10	FALSE	1.79
05	1	linear0.95	194	TRUE	4.02
05	1	linear0.95	NA	FALSE	6.68
05	1	linear0.99	10	FALSE	1.80
05	1	linear0.99	279	TRUE	4.42
05	1	linear0.99	NA	FALSE	6.74
05	1	power0.5	10	FALSE	1.65
05	1	power0.5	20	TRUE	1.81
05	1	power0.5	NA	FALSE	5.40
05	1	power0.75	10	FALSE	1.72
05	1	power0.75	39	TRUE	2.22
05	1	power0.75	NA	FALSE	5.93
05	1	power0.9	10	FALSE	1.77
05	1	power0.9	71	TRUE	2.83
05	1	power0.9	NA	FALSE	6.40
05	1	power0.95	10	FALSE	1.78
05	1	power0.95	108	TRUE	3.36
05	1	power0.95	NA	FALSE	6.57
05	1	power0.99	10	FALSE	1.79
05	1	power0.99	172	TRUE	3.94
05	1	power0.99	NA	FALSE	6.72
05	2	none	10	FALSE	1.24
05	2	none	921	TRUE	4.68
05	2	none	NA	FALSE	5.73
05	2	exponential0.5	10	FALSE	1.16
05	2	exponential0.5	68	TRUE	2.09
05	2	exponential0.5	NA	FALSE	6.58
05	2	exponential0.75	10	FALSE	1.20
05	2	exponential0.75	119	TRUE	2.78
05	2	exponential0.75	NA	FALSE	5.58

05	2	exponential0.9	10	FALSE	1.22
05	2	exponential0.9	160	TRUE	3.20
05	2	exponential0.9	NA	FALSE	5.57
05	2	exponential0.95	10	FALSE	1.23
05	2	exponential0.95	153	TRUE	3.21
05	2	exponential0.95	NA	FALSE	5.64
05	2	exponential0.99	10	FALSE	1.24
05	2	exponential0.99	157	TRUE	3.28
05	2	exponential0.99	NA	FALSE	5.71
05	2	linear0.5	10	FALSE	1.17
05	2	linear0.5	131	TRUE	2.66
05	2	linear0.5	NA	FALSE	6.62
05	2	linear0.75	10	FALSE	1.20
05	2	linear0.75	158	TRUE	3.03
05	2	linear0.75	NA	FALSE	5.60
05	2	linear0.9	10	FALSE	1.22
05	2	linear0.9	160	TRUE	3.20
05	2	linear0.9	NA	FALSE	5.57
05	2	linear0.95	10	FALSE	1.23
05	2	linear0.95	160	TRUE	3.25
05	2	linear0.95	NA	FALSE	5.64
05	2	linear0.99	10	FALSE	1.24
05	2	linear0.99	116	TRUE	2.99
05	2	linear0.99	NA	FALSE	5.71
05	2	power0.5	10	FALSE	1.12
05	2	power0.5	20	TRUE	1.32
05	2	power0.5	NA	FALSE	4.37
05	2	power0.75	10	FALSE	1.17
05	2	power0.75	40	TRUE	1.77
05	2	power0.75	NA	FALSE	4.90
05	2	power0.9	10	FALSE	1.21
05	2	power0.9	67	TRUE	2.32
05	2	power0.9	NA	FALSE	5.37
05	2	power0.95	10	FALSE	1.22
05	2	power0.95	87	TRUE	2.63
05	2	power0.95	NA	FALSE	5.55
05	2	power0.99	10	FALSE	1.23
05	2	power0.99	122	TRUE	3.03
05	2	power0.99	NA	FALSE	5.69
05	3	none	10	FALSE	1.32
05	3	none	112	TRUE	3.01
05	3	none	NA	FALSE	5.51
05	3	exponential0.5	10	FALSE	1.24
05	3	exponential0.5	64	TRUE	2.12
05	3	exponential0.5	NA	FALSE	6.28
05	3	exponential0.75	10	FALSE	1.28
05	3	exponential0.75	103	TRUE	2.70
05	3	exponential0.75	NA	FALSE	5.33
05	3	exponential0.9	10	FALSE	1.30
05	3	exponential0.9	127	TRUE	3.04
05	3	exponential0.9	NA	FALSE	5.33

05	3	exponential0.95	10	FALSE	1.31
05	3	exponential0.95	114	TRUE	2.98
05	3	exponential0.95	NA	FALSE	5.41
05	3	exponential0.99	10	FALSE	1.31
05	3	exponential0.99	144	TRUE	3.25
05	3	exponential0.99	NA	FALSE	5.49
05	3	linear0.5	10	FALSE	1.25
05	3	linear0.5	145	TRUE	2.81
05	3	linear0.5	NA	FALSE	6.32
05	3	linear0.75	10	FALSE	1.28
05	3	linear0.75	147	TRUE	3.03
05	3	linear0.75	NA	FALSE	5.34
05	3	linear0.9	10	FALSE	1.30
05	3	linear0.9	233	TRUE	3.56
05	3	linear0.9	NA	FALSE	5.34
05	3	linear0.95	10	FALSE	1.31
05	3	linear0.95	133	TRUE	3.13
05	3	linear0.95	NA	FALSE	5.41
05	3	linear0.99	10	FALSE	1.31
05	3	linear0.99	168	TRUE	3.40
05	3	linear0.99	NA	FALSE	5.49
05	3	power0.5	10	FALSE	1.16
05	3	power0.5	20	TRUE	1.38
05	3	power0.5	NA	FALSE	4.17
05	3	power0.75	10	FALSE	1.24
05	3	power0.75	39	TRUE	1.83
05	3	power0.75	NA	FALSE	4.69
05	3	power0.9	10	FALSE	1.28
05	3	power0.9	64	TRUE	2.33
05	3	power0.9	NA	FALSE	5.15
05	3	power0.95	10	FALSE	1.30
05	3	power0.95	79	TRUE	2.59
05	3	power0.95	NA	FALSE	5.33
05	3	power0.99	10	FALSE	1.31
05	3	power0.99	95	TRUE	2.83
05	3	power0.99	NA	FALSE	5.47
10	1	none	10	FALSE	1.48
10	1	none	445	TRUE	6.64
10	1	none	NA	FALSE	9.85
10	1	exponential0.5	10	FALSE	1.33
10	1	exponential0.5	80	TRUE	3.18
10	1	exponential0.5	NA	FALSE	11.15
10	1	exponential0.75	10	FALSE	1.41
10	1	exponential0.75	162	TRUE	4.61
10	1	exponential0.75	NA	FALSE	9.39
10	1	exponential0.9	10	FALSE	1.45
10	1	exponential0.9	243	TRUE	5.51
10	1	exponential0.9	NA	FALSE	9.47
10	1	exponential0.95	10	FALSE	1.46
10	1	exponential0.95	430	TRUE	6.44
10	1	exponential0.95	NA	FALSE	9.63

10	1	exponential0.99	10	FALSE	1.48
10	1	exponential0.99	749	TRUE	7.44
10	1	exponential0.99	NA	FALSE	9.80
10	1	linear0.5	10	FALSE	1.35
10	1	linear0.5	144	TRUE	4.03
10	1	linear0.5	NA	FALSE	11.23
10	1	linear0.75	10	FALSE	1.41
10	1	linear0.75	180	TRUE	4.78
10	1	linear0.75	NA	FALSE	9.43
10	1	linear0.9	10	FALSE	1.45
10	1	linear0.9	244	TRUE	5.52
10	1	linear0.9	NA	FALSE	9.47
10	1	linear0.95	10	FALSE	1.46
10	1	linear0.95	341	TRUE	6.10
10	1	linear0.95	NA	FALSE	9.63
10	1	linear0.99	10	FALSE	1.48
10	1	linear0.99	820	TRUE	7.57
10	1	linear0.99	NA	FALSE	9.81
10	1	power0.5	10	FALSE	1.26
10	1	power0.5	17	TRUE	1.49
10	1	power0.5	NA	FALSE	7.11
10	1	power0.75	10	FALSE	1.36
10	1	power0.75	35	TRUE	2.28
10	1	power0.75	NA	FALSE	8.19
10	1	power0.9	10	FALSE	1.43
10	1	power0.9	68	TRUE	3.43
10	1	power0.9	NA	FALSE	9.13
10	1	power0.95	10	FALSE	1.46
10	1	power0.95	922	TRUE	7.51
10	1	power0.95	NA	FALSE	9.49
10	1	power0.99	10	FALSE	1.47
10	1	power0.99	521	TRUE	6.84
10	1	power0.99	NA	FALSE	9.78
10	2	none	10	FALSE	1.50
10	2	none	373	TRUE	6.51
10	2	none	NA	FALSE	9.62
10	2	exponential0.5	10	FALSE	1.35
10	2	exponential0.5	72	TRUE	3.00
10	2	exponential0.5	NA	FALSE	11.40
10	2	exponential0.75	10	FALSE	1.43
10	2	exponential0.75	127	TRUE	4.31
10	2	exponential0.75	NA	FALSE	9.40
10	2	exponential0.9	10	FALSE	1.47
10	2	exponential0.9	173	TRUE	5.14
10	2	exponential0.9	NA	FALSE	9.33
10	2	exponential0.95	10	FALSE	1.49
10	2	exponential0.95	230	TRUE	5.70
10	2	exponential0.95	NA	FALSE	9.44
10	2	exponential0.99	10	FALSE	1.50
10	2	exponential0.99	263	TRUE	6.00
10	2	exponential0.99	NA	FALSE	9.58

10	2	linear0.5	10	FALSE	1.37
10	2	linear0.5	127	TRUE	3.89
10	2	linear0.5	NA	FALSE	11.47
10	2	linear0.75	10	FALSE	1.43
10	2	linear0.75	145	TRUE	4.53
10	2	linear0.75	NA	FALSE	9.43
10	2	linear0.9	10	FALSE	1.47
10	2	linear0.9	201	TRUE	5.37
10	2	linear0.9	NA	FALSE	9.33
10	2	linear0.95	10	FALSE	1.49
10	2	linear0.95	191	TRUE	5.42
10	2	linear0.95	NA	FALSE	9.44
10	2	linear0.99	10	FALSE	1.50
10	2	linear0.99	254	TRUE	5.95
10	2	linear0.99	NA	FALSE	9.58
10	2	power0.5	10	FALSE	1.28
10	2	power0.5	17	TRUE	1.51
10	2	power0.5	NA	FALSE	7.17
10	2	power0.75	10	FALSE	1.38
10	2	power0.75	38	TRUE	2.36
10	2	power0.75	NA	FALSE	8.11
10	2	power0.9	10	FALSE	1.45
10	2	power0.9	74	TRUE	3.55
10	2	power0.9	NA	FALSE	8.96
10	2	power0.95	10	FALSE	1.48
10	2	power0.95	134	TRUE	4.76
10	2	power0.95	NA	FALSE	9.28
10	2	power0.99	10	FALSE	1.50
10	2	power0.99	329	TRUE	6.30
10	2	power0.99	NA	FALSE	9.55
10	3	none	10	FALSE	1.53
10	3	none	134	TRUE	5.10
10	3	none	NA	FALSE	10.27
10	3	exponential0.5	10	FALSE	1.37
10	3	exponential0.5	63	TRUE	2.99
10	3	exponential0.5	NA	FALSE	11.72
10	3	exponential0.75	10	FALSE	1.45
10	3	exponential0.75	124	TRUE	4.47
10	3	exponential0.75	NA	FALSE	9.95
10	3	exponential0.9	10	FALSE	1.50
10	3	exponential0.9	160	TRUE	5.21
10	3	exponential0.9	NA	FALSE	9.95
10	3	exponential0.95	10	FALSE	1.52
10	3	exponential0.95	137	TRUE	5.04
10	3	exponential0.95	NA	FALSE	10.08
10	3	exponential0.99	10	FALSE	1.53
10	3	exponential0.99	147	TRUE	5.26
10	3	exponential0.99	NA	FALSE	10.23
10	3	linear0.5	10	FALSE	1.39
10	3	linear0.5	128	TRUE	4.12
10	3	linear0.5	NA	FALSE	11.80

10	3	linear0.75	10	FALSE	1.46
10	3	linear0.75	146	TRUE	4.75
10	3	linear0.75	NA	FALSE	9.99
10	3	linear0.9	10	FALSE	1.50
10	3	linear0.9	186	TRUE	5.45
10	3	linear0.9	NA	FALSE	9.96
10	3	linear0.95	10	FALSE	1.52
10	3	linear0.95	151	TRUE	5.22
10	3	linear0.95	NA	FALSE	10.09
10	3	linear0.99	10	FALSE	1.53
10	3	linear0.99	124	TRUE	4.93
10	3	linear0.99	NA	FALSE	10.23
10	3	power0.5	10	FALSE	1.29
10	3	power0.5	17	TRUE	1.52
10	3	power0.5	NA	FALSE	7.54
10	3	power0.75	10	FALSE	1.41
10	3	power0.75	33	TRUE	2.26
10	3	power0.75	NA	FALSE	8.63
10	3	power0.9	10	FALSE	1.48
10	3	power0.9	61	TRUE	3.36
10	3	power0.9	NA	FALSE	9.57
10	3	power0.95	10	FALSE	1.51
10	3	power0.95	82	TRUE	4.01
10	3	power0.95	NA	FALSE	9.91
10	3	power0.99	10	FALSE	1.53
10	3	power0.99	115	TRUE	4.78
10	3	power0.99	NA	FALSE	10.20

A.2.3 Simulation Results Summary

sim	tau	bleaching	mean_brightness	sd_brightness
01M	10	exponential0.5	0.99	0.01
01M	10	exponential0.75	1.01	0.02
01M	10	exponential0.9	1.02	0.03
01M	10	exponential0.95	1.02	0.03
01M	10	exponential0.99	1.02	0.03
01M	10	linear0.5	1.00	0.01
01M	10	linear0.75	1.01	0.02
01M	10	linear0.9	1.02	0.03
01M	10	linear0.95	1.02	0.03
01M	10	linear0.99	1.02	0.03
01M	10	none	1.02	0.03
01M	10	power0.5	0.99	0.01
01M	10	power0.75	1.00	0.02
01M	10	power0.9	1.01	0.03
01M	10	power0.95	1.02	0.03
01M	10	power0.99	1.02	0.03
01M	auto	exponential0.5	1.21	0.01
01M	auto	exponential0.75	1.40	0.01
01M	auto	exponential0.9	1.52	0.06
01M	auto	exponential0.95	1.59	0.02
01M	auto	exponential0.99	1.70	0.09
01M	auto	linear0.5	1.36	0.01
01M	auto	linear0.75	1.45	0.01
01M	auto	linear0.9	1.51	0.02
01M	auto	linear0.95	1.60	0.03
01M	auto	linear0.99	1.68	0.05
01M	auto	none	1.69	0.07
01M	auto	power0.5	1.09	0.01
01M	auto	power0.75	1.18	0.02
01M	auto	power0.9	1.26	0.02
01M	auto	power0.95	1.61	0.04
01M	auto	power0.99	1.67	0.06
01M	NA	exponential0.5	2.12	0.03
01M	NA	exponential0.75	1.93	0.06
01M	NA	exponential0.9	1.92	0.07
01M	NA	exponential0.95	1.94	0.07
01M	NA	exponential0.99	1.95	0.08
01M	NA	linear0.5	2.13	0.04
01M	NA	linear0.75	1.93	0.06
01M	NA	linear0.9	1.92	0.07
01M	NA	linear0.95	1.94	0.07
01M	NA	linear0.99	1.95	0.08
01M	NA	none	1.96	0.08
01M	NA	power0.5	1.68	0.04
01M	NA	power0.75	1.79	0.06
01M	NA	power0.9	1.88	0.07
01M	NA	power0.95	1.92	0.07
01M	NA	power0.99	1.95	0.08

05M	10	exponential0.5	1.40	0.35
05M	10	exponential0.75	1.42	0.32
05M	10	exponential0.9	1.44	0.31
05M	10	exponential0.95	1.44	0.31
05M	10	exponential0.99	1.45	0.30
05M	10	linear0.5	1.40	0.34
05M	10	linear0.75	1.42	0.32
05M	10	linear0.9	1.44	0.31
05M	10	linear0.95	1.44	0.31
05M	10	linear0.99	1.45	0.30
05M	10	none	1.45	0.30
05M	10	power0.5	1.31	0.29
05M	10	power0.75	1.38	0.30
05M	10	power0.9	1.42	0.30
05M	10	power0.95	1.44	0.30
05M	10	power0.99	1.45	0.30
05M	auto	exponential0.5	2.28	0.29
05M	auto	exponential0.75	2.96	0.38
05M	auto	exponential0.9	3.33	0.38
05M	auto	exponential0.95	3.39	0.52
05M	auto	exponential0.99	3.59	0.56
05M	auto	linear0.5	2.92	0.33
05M	auto	linear0.75	3.17	0.24
05M	auto	linear0.9	3.55	0.35
05M	auto	linear0.95	3.47	0.48
05M	auto	linear0.99	3.60	0.74
05M	auto	none	4.08	0.93
05M	auto	power0.5	1.50	0.27
05M	auto	power0.75	1.94	0.25
05M	auto	power0.9	2.50	0.29
05M	auto	power0.95	2.86	0.43
05M	auto	power0.99	3.26	0.59
05M	NA	exponential0.5	6.92	0.86
05M	NA	exponential0.75	5.88	0.75
05M	NA	exponential0.9	5.85	0.70
05M	NA	exponential0.95	5.91	0.68
05M	NA	exponential0.99	5.98	0.67
05M	NA	linear0.5	6.95	0.85
05M	NA	linear0.75	5.90	0.75
05M	NA	linear0.9	5.85	0.70
05M	NA	linear0.95	5.91	0.68
05M	NA	linear0.99	5.98	0.67
05M	NA	none	6.00	0.66
05M	NA	power0.5	4.65	0.66
05M	NA	power0.75	5.18	0.66
05M	NA	power0.9	5.64	0.67
05M	NA	power0.95	5.82	0.66
05M	NA	power0.99	5.96	0.66
10M	10	exponential0.5	1.35	0.02
10M	10	exponential0.75	1.43	0.02
10M	10	exponential0.9	1.47	0.03

10M	10	exponential0.95	1.49	0.03
10M	10	exponential0.99	1.50	0.03
10M	10	linear0.5	1.37	0.02
10M	10	linear0.75	1.43	0.02
10M	10	linear0.9	1.47	0.03
10M	10	linear0.95	1.49	0.03
10M	10	linear0.99	1.50	0.03
10M	10	none	1.50	0.03
10M	10	power0.5	1.28	0.02
10M	10	power0.75	1.38	0.02
10M	10	power0.9	1.46	0.02
10M	10	power0.95	1.48	0.03
10M	10	power0.99	1.50	0.03
10M	auto	exponential0.5	3.06	0.11
10M	auto	exponential0.75	4.46	0.15
10M	auto	exponential0.9	5.28	0.20
10M	auto	exponential0.95	5.73	0.70
10M	auto	exponential0.99	6.23	1.11
10M	auto	linear0.5	4.02	0.12
10M	auto	linear0.75	4.69	0.13
10M	auto	linear0.9	5.45	0.07
10M	auto	linear0.95	5.58	0.46
10M	auto	linear0.99	6.15	1.33
10M	auto	none	6.09	0.86
10M	auto	power0.5	1.51	0.02
10M	auto	power0.75	2.30	0.05
10M	auto	power0.9	3.45	0.10
10M	auto	power0.95	5.43	1.84
10M	auto	power0.99	5.97	1.07
10M	NA	exponential0.5	11.42	0.29
10M	NA	exponential0.75	9.58	0.32
10M	NA	exponential0.9	9.58	0.33
10M	NA	exponential0.95	9.72	0.33
10M	NA	exponential0.99	9.87	0.33
10M	NA	linear0.5	11.50	0.29
10M	NA	linear0.75	9.62	0.32
10M	NA	linear0.9	9.59	0.33
10M	NA	linear0.95	9.72	0.33
10M	NA	linear0.99	9.87	0.33
10M	NA	none	9.91	0.33
10M	NA	power0.5	7.27	0.23
10M	NA	power0.75	8.31	0.28
10M	NA	power0.9	9.22	0.31
10M	NA	power0.95	9.56	0.32
10M	NA	power0.99	9.84	0.33

A.2.4 Oligomeric State Results Within the Same Bleaching Regime

Below, mean_5mer indicates the measured fold change in brightness ϵ when the expected fold change is 5 (since we have simulated monomers and 5mers), similarly for mean_10mer; the 95% $\pm$ indicates the $\pm$ for the 95% confidence interval. For example, on row two, we have the mean fold increase for a 5mer being 4.8 ± 2.5 giving the interval $(2.3, 7.3)$..

bleaching	tau	mean_5mer	95% ±	mean_10mer	95% ±
none	10	15.0	33.0	20.3	24.5
none	auto	4.8	2.5	7.4	2.5
none	NA	5.1	1.4	9.4	1.4
exponential0.5	10	380.9	2302.5	682.3	1414.0
exponential0.5	auto	5.7	2.5	9.9	1.5
exponential0.5	NA	5.0	1.3	9.3	0.7
exponential0.75	10	24.3	59.0	30.4	36.7
exponential0.75	auto	4.4	1.6	8.6	0.7
exponential0.75	NA	5.0	1.5	9.2	1.1
exponential0.9	10	17.3	38.9	22.5	26.8
exponential0.9	auto	4.5	1.6	8.1	1.9
exponential0.9	NA	5.0	1.4	9.3	1.3
exponential0.95	10	16.0	35.4	21.2	25.3
exponential0.95	auto	3.7	1.5	7.9	2.1
exponential0.95	NA	5.0	1.4	9.4	1.3
exponential0.99	10	15.2	33.3	20.4	24.5
exponential0.99	auto	3.6	1.6	7.4	3.2
exponential0.99	NA	5.1	1.4	9.4	1.4
linear0.5	10	73.3	189.0	83.1	97.4
linear0.5	auto	5.0	1.6	8.4	0.6
linear0.5	NA	5.0	1.3	9.3	0.7
linear0.75	10	22.8	53.9	28.3	33.5
linear0.75	auto	4.6	0.9	8.3	0.5
linear0.75	NA	5.0	1.5	9.2	1.2
linear0.9	10	17.4	38.8	22.6	26.8
linear0.9	auto	4.9	1.2	8.5	0.7
linear0.9	NA	5.0	1.4	9.3	1.3
linear0.95	10	16.2	36.3	21.5	26.1
linear0.95	auto	3.8	1.4	7.4	1.5
linear0.95	NA	5.0	1.4	9.4	1.3
linear0.99	10	15.2	33.3	20.4	24.5
linear0.99	auto	3.5	1.9	7.2	3.5
linear0.99	NA	5.1	1.4	9.4	1.4
power0.5	10	107.9	377.8	180.7	19.8
power0.5	auto	4.2	5.1	5.6	1.4
power0.5	NA	5.0	1.7	9.2	1.1
power0.75	10	99.3	534.3	187.4	393.3
power0.75	auto	4.9	2.6	7.6	1.5
power0.75	NA	5.0	1.6	9.3	1.3
power0.9	10	19.4	51.4	27.1	37.2
power0.9	auto	5.5	2.0	9.5	1.3
power0.9	NA	5.0	1.5	9.4	1.4
power0.95	10	17.0	40.9	23.3	30.1
power0.95	auto	2.8	1.3	6.5	5.2

power0.95	NA	5.1	1.4	9.4	1.4
power0.99	10	15.3	34.2	20.7	25.3
power0.99	auto	3.1	1.6	7.9	2.9
power0.99	NA	5.1	1.4	9.4	1.4

A.3 Reproducibility

All of the files and scripts used to perform the analyses used in this paper can be found at https://github.com/rorynolan/nandb/tree/master/paper_data. To reproduce any of the analyses, you must first download this paper_data folder. This can be downloaded as a zip file from the web or alternatively it is possible to clone the whole https://github.com/rorynolan/nandb repository (if you don't know what this means, search the web for "how to clone a Github repository"). Below it is assumed that you have R, Rstudio and the nandb package installed.

A.3.1 Reproducing the Main Figure in the Paper

From the paper_data/ folder, navigate to the mainfig/ folder. Open the mainfig.Rproj file by double-clicking it. Within Rstudio, open the mainfig.R file and run all of the code therein. The script only uses the .tif files (the raw data) and produces everything else.

- The output file fig.pdf is the main figure.

A.3.2 Reproducing the FKBP Data

From the paper_data/ folder, navigate to the fkbp/ folder. Open the fkbp.Rproj file by double-clicking it. Within Rstudio, open the fkbp.R file and run all of the code therein. The script only uses the .tif files (the raw data) and produces everything else.

- The output file master.csv is the table in section A.1.1.
- The output file compareconcs.pdf is figure S3.
- The output file fold_changes_cells.csv is (a reformatted version of) the table in section A.1.2.
- The output file fkbp_median_summary.csv is table S1.
- The output file foldchanges.pdf is figure S4.

A.3.3 Reproducing the Simulated Data

From the paper_data/ folder, navigate to the sim/ folder. Open the sim.Rproj file by double-clicking it. Within Rstudio, open the sim.R file and run all of the code therein. The script only uses the Simulation[m]_rep[n].tif files (where [m] and [n] are integers, i.e. the files that were simulated with SimFCS [7] and don't include bleaching) and produces everything else (including the .tif files that include a bleaching regime).

- The output file master.csv is the table in section A.2.2.
- The output file olig.csv is the table in section A.2.4.
- The output file fold_change_mix.csv is table S2.
- The output file clust.csv is the table in section A.2.3.
- The output file clust.pdf is figure S6.

A.3.4 Reproducing the Figure for Visualising Exponential Smoothing Edge Correction (section 1.3.3)

From the paper_data/ folder, navigate to the edges/ folder. Open the edges.Rproj file by double-clicking it. Within Rstudio, open the edges.R file and run all of the code therein.

- The output file dontbenaive.pdf is figure S1.

A.3.5 Reproducing the Data for Visualising Bleaching Correction (section 8)

From the `paper_data/` folder, navigate to the `bleachvis/` folder. Open the `bleachvis.Rproj` file by double-clicking it. Within Rstudio, open the `bleachvis.R` file and run all of the code therein. The script only uses the `.tif` file (the raw data) and produces everything else.

- The output files `bleachvis1.pdf` and `bleachvis2.pdf` are figures S7 and S8.

References

[1] P. D. Stroud, "A recursive exponential filter for time-sensitive data," *Los Alamos National Laboratory*, 1999.

[2] A. J. Lam, F. St-Pierre, Y. Gong, J. D. Marshall, P. J. Cranfill, M. A. Baird, M. R. McKeown, J. Wiedenmann, M. W. Davidson, M. J. Schnitzer, R. Y. Tsien, and M. Z. Lin, "Improving FRET dynamic range with bright green and red fluorescent proteins," *Nat. Methods*, vol. 9, no. 10, pp. 1005–1012, Oct 2012.

[3] S. Maurer-Stroh, B. Eisenhaber, and F. Eisenhaber, "N-terminal N-myristoylation of proteins: prediction of substrate proteins from amino acid sequence," *J. Mol. Biol.*, vol. 317, no. 4, pp. 541–557, Apr 2002.

[4] A. R. Aricescu, W. Lu, and E. Y. Jones, "A time- and cost-efficient system for high-level protein production in mammalian cells," *Acta Crystallogr. D Biol. Crystallogr.*, vol. 62, no. Pt 10, pp. 1243–1250, Oct 2006.

[5] S. Mangiafico, *rcompanion: Functions to Support Extension Education Program Evaluation*, 2017, r package version 1.5.6. [Online]. Available: https://CRAN.R-project.org/package=rcompanion

[6] T. Clackson, W. Yang, L. W. Rozamus, M. Hatada, J. F. Amara, C. T. Rollins, L. F. Stevenson, S. R. Magari, S. A. Wood, N. L. Courage, X. Lu, F. Cerasoli, M. Gilman, and D. A. Holt, "Redesigning an FKBP-ligand interface to generate chemical dimerizers with novel specificity," *Proc. Natl. Acad. Sci. U.S.A.*, vol. 95, no. 18, pp. 10 437–10 442, Sep 1998.

[7] L. for Fluorescence Dynamics, *Globals for Images: SimFCS 4*. [Online]. Available: http://www.lfd.uci.edu/globals/

[8] N. Bag and T. Wohland, "Imaging fluorescence fluctuation spectroscopy: new tools for quantitative bioimaging," *Annu Rev Phys Chem*, vol. 65, pp. 225–248, 2014.

[9] Leica, *Simple Number and Brightness Analysis with the Leica TCS SP5 and HyD Detection.*

[10] Zeiss, *ZEN Black*. [Online]. Available: https://www.zeiss.com/microscopy/int/products/microscope-software.html
